# Aging brain shows joint declines in brain within-network connectivity and between-network connectivity: a large-sample study (*N* > 6,000)

**DOI:** 10.3389/fnagi.2023.1159054

**Published:** 2023-05-18

**Authors:** Yuhui Du, Yating Guo, Vince D. Calhoun

**Affiliations:** ^1^School of Computer and Information Technology, Shanxi University, Taiyuan, China; ^2^Tri-Institutional Center for Translational Research in Neuroimaging and Data Science (TReNDS), Georgia Institute of Technology, Georgia State University, Emory University, Atlanta, GA, United States

**Keywords:** brain functional network, functional network connectivity, normal brain aging, joint changes, NeuroMark

## Abstract

**Introduction:**

Numerous studies have shown that aging has important effects on specific functional networks of the brain and leads to brain functional connectivity decline. However, no studies have addressed the effect of aging at the whole-brain level by studying both brain functional networks (i.e., within-network connectivity) and their interaction (i.e., between-network connectivity) as well as their joint changes.

**Methods:**

In this work, based on a large sample size of neuroimaging data including 6300 healthy adults aged between 49 and 73 years from the UK Biobank project, we first use our previously proposed priori-driven independent component analysis (ICA) method, called NeuroMark, to extract the whole-brain functional networks (FNs) and the functional network connectivity (FNC) matrix. Next, we perform a two-level statistical analysis method to identify robust aging-related changes in FNs and FNCs, respectively. Finally, we propose a combined approach to explore the synergistic and paradoxical changes between FNs and FNCs.

**Results:**

Results showed that the enhanced FNCs mainly occur between different functional domains, involving the default mode and cognitive control networks, while the reduced FNCs come from not only between different domains but also within the same domain, primarily relating to the visual network, cognitive control network, and cerebellum. Aging also greatly affects the connectivity within FNs, and the increased within-network connectivity along with aging are mainly within the sensorimotor network, while the decreased within-network connectivity significantly involves the default mode network. More importantly, many significant joint changes between FNs and FNCs involve default mode and sub-cortical networks. Furthermore, most synergistic changes are present between the FNCs with reduced amplitude and their linked FNs, and most paradoxical changes are present in the FNCs with enhanced amplitude and their linked FNs.

**Discussion:**

In summary, our study emphasizes the diversity of brain aging and provides new evidence via novel exploratory perspectives for non-pathological aging of the whole brain.

## 1. Introduction

As the world is entering a rapidly aging society (Beard et al., 2016), the problem of population aging has raised widespread concern. Aging is often accompanied by progressive degeneration of all organs of the body, particularly the brain, which affects memory, learning, and other cognitive functions (Naik et al., 2017). There are thus increasing interests in studying the mechanisms of brain aging, which may provide evidence to help facilitate healthy aging. Neuroimaging techniques such as functional magnetic resonance imaging (fMRI) can help us to further disclose the influence of aging on the brain function.

An increasing number of studies have shown that aging has a direct effect on the functional network and connectivity of the brain (Sala-Llonch et al., 2015; Edde et al., 2020). However, different analysis methods could result in various or even disparate findings (Jockwitz and Caspers, 2021). For example, one previous study (Zonneveld et al., 2019) based on independent component analysis (ICA) (Calhoun and de Lacy, 2017) found that the connectivity is enhanced in older individuals within the visual domain, while an opposite conclusion was drawn using graph theory analysis (Xiang et al., 2020) in a paper (Stumme et al., 2020) that supports the connectivity is decreased with aging within visual domain. It is well acknowledged that an advanced analysis method can play an important role in maximizing the reliability of findings in terms of brain function aging.

Researchers often apply region-of-interest (ROI) based methods (Cansino, 2022), ICA, or graph theory technique to extract and analyze brain functional network and connectivity. However, ROI based methods depend heavily on the definition or selection of prior brain regions, thus such approaches may be most useful to detect brain aging effects on targeted specific functional networks and connectivity (Zhang et al., 2014; Hausman et al., 2020). Although there have been some studies that apply whole-brain ROIs to explore aging effects on brain functional connectivity at the whole brain level (King et al., 2018), they only focus on the connectivity between ROIs but cannot investigate the changes within ROIs. Different from the ROI based method, ICA is data-driven as there is no need to define brain regions in advance based on subjective knowledges. More importantly, ICA can output spatial functional networks as well as the temporal fluctuations of functional networks, naturally providing a chance to study brain functional networks and meanwhile their interaction relationship. Regarding the spatial independent components obtained from ICA on fMRI data, each biologically meaningful component reflects one brain functional network in which the Z-score of each voxel represents its within-network connectivity extent. In addition to the components, the estimated time courses from ICA include temporal information of functional networks, so they are often used to further compute functional connectivity between those networks. Previous aging-related studies using ICA only worked on some spatial functional networks (Ge et al., 2014) or functional connectivity between specific networks (Jockwitz et al., 2017; Patil et al., 2021). There has also been some research work exploring whole brain functional network connectivity using graph theory approaches (Geerligs et al., 2015; Varangis et al., 2019; Stumme et al., 2020), which analyzed the data in a more integrative way, but primarily focused only on functional connectivity. To the best of our knowledge, most previous studies using ICA only explored the within-network connectivity or between-network connectivity to understand the brain aging mechanism, and few studies investigated both within-network and between-network connectivity in a unified framework, furthermore the joint changes between within-network and between-network connectivity along with aging are largely unknown.

Data quality and sample size can impact the effectiveness of brain aging exploration. However, many studies used relatively small sample numbers, resulting in less generalizable findings. For example, a study comprising 40 old adults (aged 59–74 years) and 40 young adults (aged 18–26 years; Linda et al., 2015) demonstrated that local efficiency of sensorimotor network does not change significantly. However, this is inconsistent with another study that showed increased local efficiency of sensorimotor network using 26 young adults (aged 21–28 years) and 24 old adults (aged 51–65 years; Song et al., 2014). Using 114 subjects aged 48–89 years, another study reported decreased connectivity strength for subjects aged 65–79 years (Farràs-Permanyer et al., 2019), and did not detect progressive aging. In addition to the data quantity, data quality can also affect the reliability of the results. One previous study used two datasets, young and old subjects, with a sample size of 50, and the data age range was 21–28 for young subjects and 52–64 for old subjects (Song et al., 2014). Another study used three sets including young adults of 18–29 years, middle-aged adults of 43–55 years and old adults of 63–76 years (King et al., 2018). Although many studies have attempted to cover young and older adults, the age ranges were often determined subjectively and did not consider the sample balance among different ages. And, some studies involved data with an imbalance ratio between the males and females (Hughes et al., 2020; Stumme et al., 2020), however males and females can present different brain aging mechanisms (Foo et al., 2021; Ficek-Tani et al., 2022), which could influence findings. In summary, lower data quantity and quality of participants such as imbalanced sample number across different age groups and between females and males, small sample size, and large age span may not support a more in-depth exploration of brain aging, therefore, strict quality control of data as well as large-sample data would be beneficial for a more reliable finding.

To address the above issues, in this paper we employ our previously proposed NeuroMark method (Du et al., 2020) to obtain both the whole-brain spatial functional networks and their temporal connectivity relations so as to explore the aging effect at both the within-network and between-network levels. To maximize the reliability of the findings, we leverage a large sample size of fMRI data from 6,300 healthy subjects that almost include all available subjects aged between 49 and 73 years in the UK Biobank project (Sudlow et al., 2015) for the exploration of brain changes along with the aging. To avoid false positives in the results, we exactly match the number of subjects between gender and across different ages. By making full use of the within-network connectivity and the between-network connectivity, our study not only aims to reveal how each network changes, but also expects to disclose how the interaction between different networks in various functional domains declines along with the aging process. After obtaining whole-brain spatial functional networks and their connectivity via NeuroMark, we propose a two-level statistical analysis method to detect how aging progressively affects the brain and to identify robust aging-related brain alterations. Since NeuroMark used in this paper simultaneously yields large-scale brain functional networks and their functional connectivities, there is a unique advantage to investigate their systematical changes. In this paper, we propose novel fusion analysis strategies to investigate systematical changes between the within-network connectivity and between-network connectivity as people age.

## 2. Materials and methods

### 2.1. Subjects

To study the aging effect on brain function, we use a large sample size of resting-state fMRI data that covers most available healthy subjects aged from 49 to 73 years that participate in brain fMRI scanning in the UK Biobank project (Sudlow et al., 2015). The UK Biobank project is a prospective cohort study with deep genetic and phenotypic data collected on approximately 500,000 individuals from across the United Kingdom. The subjects who had any mental, neural system, and other diseases that could affect the brain function are discarded. In detail, we excluded the subjects with the following diseases diagnosed by ICD-10: malignant neoplasms of eye, brain, and other parts of the central nervous system, mental and behavioral disorders, diseases of the nervous system, diseases of the eye and adnexa, diseases of the ear and mastoid process, cerebrovascular diseases, and congenital malformations of the nervous system. Discovering the neural changes linked to progressive aging in brain is our primary interest, so we strictly balance the number of subjects at each age to be the same. In the UK Biobank project, there are only a few subjects under 49 years old and over 73 years old that were scanned for collecting fMRI data, so we study the subjects aged from 49 to 73 years in order to maintain more subjects for each age group as well as cover the main aging period (Peters, 2006). Similarly, the number of females and males is set to be identical for each age group, which maximizes the reliability of our findings in the general population. After performing the quality controls on the preprocessed fMRI data to select the data with slight head motion (described in Section 2.2), the remaining subjects are divided into 25 groups according to their age, while each of the 25 age groups includes 252 subjects (126 females, 126 males), resulting in n=6,300 subjects (mean age: 61, males: 3150) for our study.

### 2.2. Image acquisition and preprocessing

All participants underwent resting-state fMRI scanning performed on a Siemens Skyra 3T scanner (Siemens Medical Solutions, Erlangen, Germany). FMRI data was obtained using a blood-oxygenation level dependent (BOLD) with an echo-planar imaging (EPI) sequence (TR = 0.735 s, TE = 39 ms, FoV =88 × 88 × 64, voxel resolution 2.4 × 2.4 × 2.4 mm, flip angle = 52^o^), lasting for 6 min for 490 time points. Participants were instructed to relax and think of nothing while focusing their eyes on a crosshair during the scanning.

For all subjects with fMRI data in the UK Biobank project, we preprocess their fMRI data using the statistical parametric mapping (SPM) software (Jia et al., 2019). For each subject’s data, after discarding the first ten image volumes, we perform the slice-timing and motion correction, normalization into the Montreal Neurological Institute (MNI) space using the echo-planar imaging (EPI) template, data resampling to 3 × 3 × 3 mm^3^ isotropic voxels, and finally spatial smoothing using a Gaussian function with a specific width at half of the maximum value (FWHM) = 6 mm. After that, we only preserve the subject data with slight head motion. As such, all remaining data has head motion with less than 3.0mm x , y , and z translations and less than 3° pitch, yaw, and roll rotations, measured by the averaged head motion value across all time points.

### 2.3. Computation of brain functional networks and connectivity via a NeuroMark method

In order to comprehensively explore brain function alterations along with the aging process, we apply our previously proposed NeuroMark method (Du et al., 2020) to obtain both brain functional networks and the connectivity between networks. NeuroMark computes reliable functional network templates based on two independent resting-state fMRI datasets of healthy controls from the Brain Genomics Superstruct Project (GSP; Holmes et al., 2015) and the Human Connectome Project (HCP; Van Essen et al., 2013). We first decompose these two datasets separately using standard group-level ICA to identify 100 independent components (ICs) for each dataset. The Supplementary Figures S1–S3 show the spatial maps of the 100 ICs obtained from the GSP dataset, which 3D image data can be downloaded from www.yuhuidu.com. Next, we match the two sets of ICs (from GSP and HCP) using a greedy spatial correlation analysis to determine a set of reproducible and meaningful ICs as the reliable network templates. Specifically, IC pairs are regarded as reproducible if they show a higher spatial correlation than 0.4. From the reproducible (i.e., highly matched) ICs, 53 pairs of ICs are regarded as brain functional networks and then are assigned to different functional domains. Rather than averaging each pair of ICs to estimate one network template, we select the 53 ICs from the GSP dataset as 53 functional network templates, because they are smoother with less noises than that from HCP. After that, through an advanced multi-objective optimization-based ICA, NeuroMark automatically extracts accurate large-scale networks based on the 53 reliable functional network templates in a data driven manner (Du and Fan, 2013). The NeuroMark toolbox and codes are freely accessible on www.yuhuidu.com. The NeuroMark method has been widely applied to various studies in the neuroscience field, such as extracting the brain dynamics of different sites to explore its abnormalities of patients with schizophrenia, bipolar and schizoaffective disorders (Sendi et al., 2022; Gazula et al., 2023), investigating the commonality and uniqueness between autism spectrum disorder and schizophrenia (Du et al., 2021, 2022), and exploring differences between genders using functional networks (Dhamala et al., 2023). Guided by 53 reliable functional network templates, in the study the corresponding 53 functional networks are computed for each of 6,300 subjects based on the preprocessed fMRI data. Because the 53 functional network templates are arranged into seven functional domains according to their functional and anatomical roles (Allen et al., 2014), including the subcortical (SC), auditory (AU), sensorimotor (SM), visual (VI), cognitive control (CC), default mode (DM), and cerebellar (CB) domains, the individual-level functional networks also naturally belong to these domains, which enables the subsequent investigation in terms of the aging affects at the functional domain level. Regarding those 53 network templates, the detailed component IDs and the peak Z-scores’ coordinates as well as their domain labels are provided in Table 1. After obtaining functional networks of all subjects by the NeuroMark, we also use the head motion parameters summarized by mean translation and rotation to regress out the motion effect from each voxel’s value in functional networks. Particularly, we averaged the absolute translation values and the absolute rotation values across all time points, respectively, to obtain the mean translation and the mean rotation values for each subject, then took the two metrices as two covariates to regress out their effects from each voxel’s Z-score in each network for all subjects. As such, the head motion’s negative effects should have been well removed and did not greatly affect the findings.

**Table 1:** Peak coordinates of functional network templates in NeuroMark.

FNs (ID)	*X*	*Y*	*Z*	FNs (ID)	*X*	*Y*	*Z*
**Sub-cortical (SC) domain**				**Cognitive-control (CC) domain**			
Caudate (69)	6.5	10.5	5.5	Inferior parietal lobule ([IPL], 68)	45.5	−61.5	43.5
Subthalamus/hypothalamus (53)	−2.5	−13.5	−1.5	Insula (33)	−30.5	22.5	−3.5
Putamen (98)	−26.5	1.5	−0.5	Superior medial frontal gyrus ([SMFG], 43)	−0.5	50.5	29.5
Caudate (99)	21.5	10.5	−3.5	Inferior frontal gyrus ([IFG], 70)	−48.5	34.5	−0.5
Thalamus (45)	−12.5	−18.5	11.5	Right inferior frontal gyrus ([R IFG], 61)	53.5	22.5	13.5
**Auditory (AU) domain**				Middle frontal gyrus ([MiFG], 55)	−41.5	19.5	26.5
Superior temporal gyrus ([STG], 21)	62.5	−22.5	7.5	Inferior parietal lobule ([IPL], 63)	−53.5	−49.5	43.5
Middle temporal gyrus ([MTG], 56)	−42.5	−6.5	10.5	Right inferior parietal lobue ([R IPL], 79)	44.5	−34.5	46.5
**Sensorimotor (SM) domain**				Supplementary motor area ([SMA], 84)	−6.5	13.5	64.5
Postcentral gyrus ([PoCG], 3)	56.5	−4.5	28.5	Superior frontal gyrus ([SFG], 96)	−24.5	26.5	49.5
Left postcentral gyrus ([L PoCG], 9)	−38.5	−22.5	56.5	Middle frontal gyrus ([MiFG], 88)	30.5	41.5	28.5
Paracentral lobule ([ParaCL], 2)	0.5	−22.5	65.5	Hippocampus ([HiPP], 48)	23.5	−9.5	−16.5
Right postcentral gyrus ([R PoCG], 11)	38.5	−19.5	55.5	Left inferior parietal lobue ([L IPL], 81)	45.5	−61.5	43.5
Superior parietal lobule ([SPL], 27)	−18.5	−43.5	65.5	Middle cingulate cortex ([MCC], 37)	−15.5	20.5	37.5
Paracentral lobule ([ParaCL], 54)	−18.5	−9.5	56.5	Inferior frontal gyrus ([IFG], 67)	39.5	44.5	−0.5
Precentral gyrus ([PreCG], 66)	−42.5	−7.5	46.5	Middle frontal gyrus ([MiFG], 38)	−26.5	47.5	5.5
Superior parietal lobule ([SPL], 80)	20.5	−63.5	58.5	Hippocampus ([HiPP], 83)	−24.5	−36.5	1.5
Postcentral gyrus ([PoCG], 72)	−47.5	−27.5	43.5	**Default-mode (DM) domain**			
**Visual (VI) domain**				Precuneus (32)	−8.5	−66.5	35.5
Calcarine gyrus ([CalcarineG], 16)	−12.5	−66.5	8.5	Precuneus (40)	−12.5	−54.5	14.5
Middle occipital gyrus ([MOG], 5)	−23.5	−93.5	−0.5	Anterior cingulate cortex ([ACC], 23)	−2.5	35.5	2.5
Middle temporal gyrus ([MTG], 62)	48.5	−60.5	10.5	Posterior cingulate cortex ([PCC], 71)	−5.5	−28.5	26.5
Cuneus (15)	15.5	−91.5	22.5	Anterior cingulate cortex ([ACC], 17)	−9.5	46.5	−10.5
Right middle occipital gyrus ([R MOG], 12)	38.5	−73.5	6.5	Precuneus (51)	−0.5	−48.5	49.5
Fusiform gyrus (93)	29.5	−42.5	−12.5	Posterior cingulate cortex ([PCC], 94)	−2.5	54.5	31.5
Inferior occipital gyrus ([IOG], 20)	−36.5	−76.5	−4.5	**Cerebellar (CB) domain**			
Lingual gyrus ([LingualG], 8)	−8.5	−81.5	−4.5	Cerebellum ([CB], 13)	−30.5	−54.5	−42.5
Middle temporal gyrus ([MTG], 77)	−44.5	−57.5	−7.5	Cerebellum ([CB], 18)	−32.5	−79.5	−37.5
				Cerebellum ([CB], 4)	20.5	−48.5	−40.5
				Cerebellum ([CB], 7)	30.5	−63.5	−40.5

The IC ID in each bracket corresponds to the index of IC in the 100 ICs obtained from the GSP dataset.

In addition to the spatial functional networks, we also compute the time courses (TCs) of those networks to reflect their temporal information using the NeuroMark method. After obtaining the TCs of all functional networks, we perform post-processing on each TC, including transforming the TC to a Fisher’s Z-score, using the six head motion parameters to regress TC, removing linear trends and spikes, and filtering via a band-pass filter with [0.01–0.15] Hz. Next, a functional network connectivity (FNC) matrix is obtained by calculating Pearson correlations between the processed TCs of those functional networks to reflect the interaction among networks. After obtaining the FNC matrices of all subjects, we also further remove the motion effect from each FNC by regressing out the head motion summarized by the mean translation and rotation values.

While the spatial map of each functional network reflects the within-network connectivity (Du et al., 2015), the FNC matrix reflects the complex interaction relationship between different networks (Du et al., 2020). By making full use of the within-network connectivity and the between-network connectivity, our study aims to reveal how the within-network and between-network connectivity decline along with the aging process, and more importantly also focused on the systematical changes between the within-network connectivity and the between-network connectivity as people age.

### 2.4. Investigation of joint changes in brain functional networks and functional network connectivity

In this section, we first clarify how we investigate the changes for the FNC and functional networks separately, and then describe how we identify their joint changes along with the aging.

#### 2.4.1. Investigating aging effect on brain functional network connectivity

**Figure 1:**
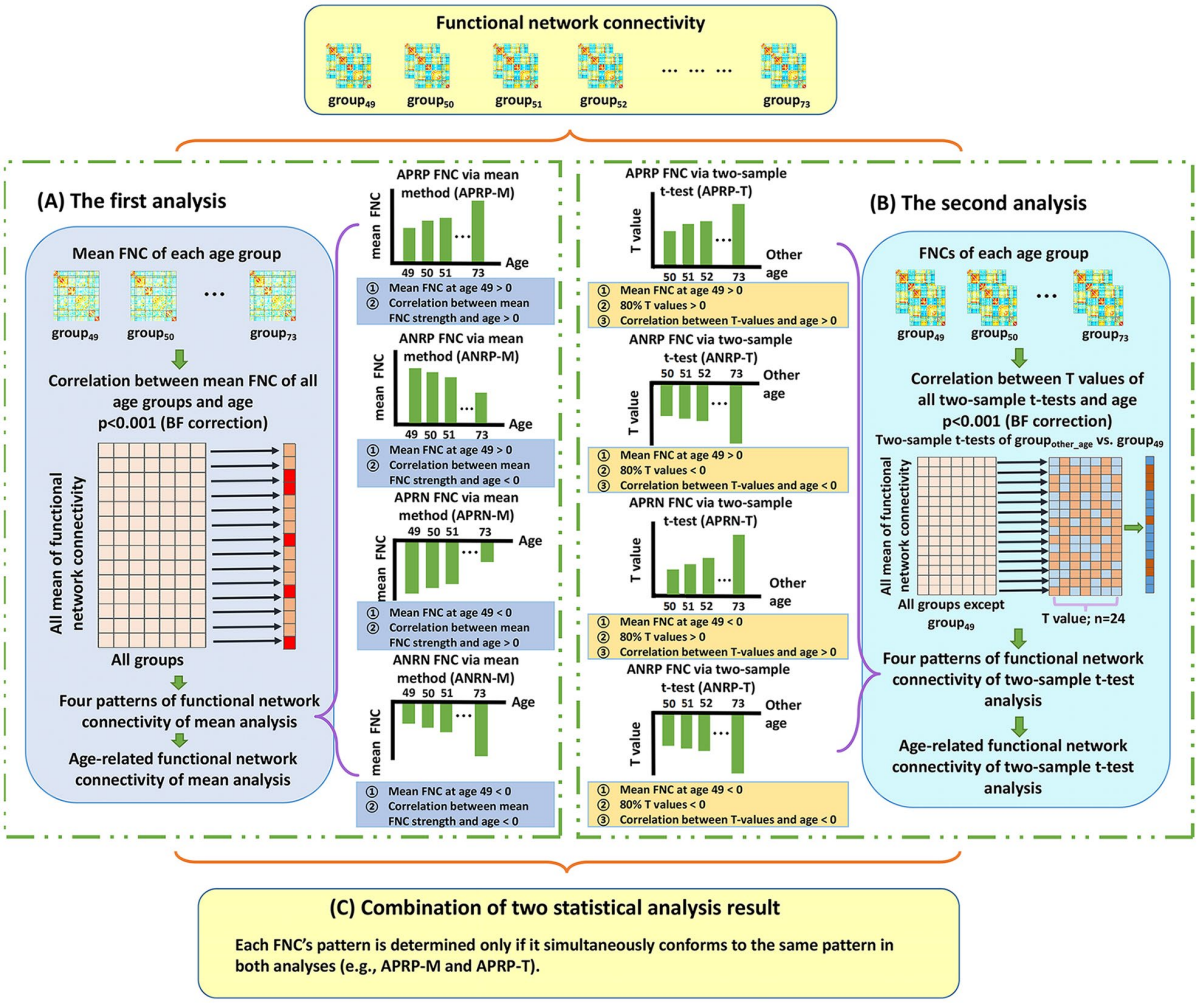
The whole two-level analysis framework of functional network connectivity (FNC). **(A)** The first analysis. For each FNC, we compute mean FNC strength in each age group and then compute Pearson correlation between the mean FNC strengths of all age groups and the ages. After that, each significant age-related FNC passing the correction [*p* < 0.001, Bonferroni (BF) correction] is assessed to judge if it belongs to one of four patterns. The defined four patterns include the age-positively-related positive FNC (APRP-M), the age-negatively-related positive FNC (ANRP-M), the age-positively-related negative FNC (APRN-M), and the age-negatively-related negative FNC (ANRN-M). **(B)** The second analysis. For each FNC, we perform two-tailed two-sample *t*-test analysis on FNC between each other age group and the group of age 49, and then compute Pearson correlation between the resulting *T*-values and corresponding ages. After that, each significant age-related FNC passing the correction [*p* < 0.001, Bonferroni (BF) correction] is assessed to judge if it belongs to one of four patterns including the age-positively-related positive FNC (APRP-T), the age-negatively-related positive FNC (ANRP-T), the age-positively-related negative FNC (APRN-T), and the age-negatively-related negative FNC (ANRN-T). **(C)** The combination of results of two statistical analyses. Reliable age-related FNC are identified only if it is significantly associated with age and belongs to the same pattern in both analyses. So, four patterns of age-related FNC are finally summarized, including the APRP, ANRP, APRN, and ANRN patterns.

Here, we first introduce the analysis procedure on the FNCs, because the connectivity number in the FNC matrix is much lower than the voxel number in the functional networks. To maximize the reliability in identifying the aging-related FNCs, we propose a two-level statistical analysis framework which idea is that two types of statistical analysis are separately performed to find the age-related FNCs and then the significant FNCs in both analyses are taken as reliable aging-related FNCs. Figure 1 shows the analysis framework on the FNCs.

In the first analysis (Figure 1A), for each FNC, we calculate its mean strength across subjects in each age group (e.g., the group that include subjects at age 70: group_70_), and then compute Pearson correlation (denoted by $r_{\mathrm{FNCMean}\_\mathrm{Age}}$ ) between the mean FNC strength values from different age groups (i.e., group_49_ to group_73_) and the ages (i.e., 49–73) to evaluate if the FNC is significantly associated with the aging process [*p* < 0.001, Bonferroni (BF) correction]. Next, each significant age-related FNC surviving BF correction is assessed to determine if it belongs to one of four patterns that are defined according to the mean FNC strength of group_49_ as well as the correlation between mean FNC and age (i.e., $r_{\mathrm{FNCMean}\_\mathrm{Age}}$ ). Here, the mean FNC strength of group_49,_ as a baseline, is used to separate positive and negative FNCs. As such, in the first analysis using the mean FNC strength, the defined four patterns include the age-positively-related positive FNC (APRP-M), the age-negatively-related positive FNC (ANRP-M), the age-positively-related negative FNC (APRN-M), and the age-negatively-related negative FNC (ANRN-M). Taking FNC belonging to the APRP-M pattern as an example, its mean FNC strength of group_49_ is positive, and the mean FNC strength across different ages has a positive correlation with age (i.e., $r_{\mathrm{FNCMean}\_\mathrm{Age}}$ > 0). Similarly, regarding the ANRP-M pattern, its mean FNC strength of group_49_ is positive, and the mean FNC strength across different ages has a negative correlation with age (i.e., $r_{\mathrm{FNCMean}\_\mathrm{Age}}$ < 0). Regarding the APRN-M pattern, its mean FNC strength of group_49_ is negative, and the mean FNC strength across different ages has a positive correlation with age (i.e., $r_{\mathrm{FNCMean}\_\mathrm{Age}}$ > 0). And regarding the ANRN-M pattern, its mean FNC strength of group_49_ is negative, and the mean FNC strength across different ages has a negative correlation with age (i.e., $r_{\mathrm{FNCMean}\_\mathrm{Age}}$ < 0).

In the second analysis (Figure 1B), for each FNC, we first perform a two-tailed two-sample *t*-test between its strength of group_other-age_ (e.g., group_50_) and that of the group_49_, and then utilize 24 *T*-values of all two-sample *t*-tests to calculate its Pearson correlation (denoted by $r_{\mathrm{FNCT}\_\mathrm{Age}}$ ) with the ages (i.e., from 50 to 73). In this paper, we use the group aged 49 as the baseline for conducting the two-sample *t*-tests, with an expectation of investigating the progressive aging path. Next, the FNCs that are significantly associated with aging according to $r_{\mathrm{FNCT}\_\mathrm{Age}}$ (*p* < 0.001, BF correction) are evaluated using our defined four patterns. The four patterns are defined based on the mean FNC strength at group_49_, the *T*-values from the between-group two-sample *t*-tests, and the correlation $r_{\mathrm{FNCT}\_\mathrm{Age}}$ . As such, for the second analysis using *T*-values, the four patterns include the age-positively-related positive FNC (APRP-T), the age-negatively-related positive FNC (ANRP-T), the age-positively-related negative FNC (APRN-T), and the age-negatively-related negative FNC (ANRN-T). In particular, an FNC is considered to belong to the APRP-T pattern if the mean FNC strength of group_49_ is positive, more than 80% *T*-values are positive, and the *T*-values have a positive correlation with the ages ( $r_{\mathrm{FNCT}\_\mathrm{Age}}$ > 0). For the ANRP-T pattern, the mean strength of FNC of group_49_ is positive, more than 80% *T*-values are negative, and the *T*-values have a negative correlation with the ages ( $r_{\mathrm{FNCT}\_\mathrm{Age}}$ < 0). Regarding the APRN-T pattern, the mean FNC strength of group_49_ is negative, more than 80% *T*-values are positive, and the *T*-values have a positive correlation with the ages ( $r_{\mathrm{FNCT}\_\mathrm{Age}}$ > 0). Regarding the APRN-T pattern, similarly, the mean FNC strength of group_49_ is negative, more than 80% *T*-values are negative, and the *T*-values have a negative correlation with the ages ( $r_{\mathrm{FNCT}\_\mathrm{Age}}$ < 0). Moreover, we summarize the number of FNCs that significantly show group difference (*p* < 0.01) in two-sample *t*-tests between group_other-age_ and group_49_ to investigate whether the difference increases along with the increasing age gap.

Based on the above-mentioned two-level analyses, we summarize the results to obtain reliable age-related FNCs (Figure 1C). Here, each FNC’s pattern is determined only if it belongs to the same pattern in both analyses. For example, if one FNC belongs to both APRP-M and APRP-T, it is regarded as belonging to the APRP pattern. So, four patterns of age-related FNC are finally summarized including the age-positively-related positive (APRP) FNC, the age-negatively-related positive (ANRP) FNC, the age-positively-related negative (APRN) FNC, and the age-negatively-related negative (ANRN) FNC. Then, its mean correlation from the two analyses is computed by averaging $r_{\mathrm{FNCMean}\_\mathrm{Age}}$ and $r_{\mathrm{FNCT}\_\mathrm{Age}}$ to reflect its association with aging, and its statistical significance is obtained by averaging $p_{\mathrm{FNCMean}\_\mathrm{Age}}$ and $p_{\mathrm{FNCT}\_\mathrm{Age}}$ .

Furthermore, we summarize the properties of reliable age-related FNCs for each pattern. Since each FNC links two functional networks and each functional network is assigned to one functional domain, we investigate FNC-linked functional domains to assess if different changing patterns show different within-domain and between-domain connectivity properties. Here, one FNC is taken as the within-domain only when two functional networks belong to the same functional domain (e.g., DM), or it is taken as the between-domain (e.g., one network belongs to the DM and the other one belongs to the VI). For this goal, we sum up the number of FNCs that each functional domain involves for each changing pattern separately in order to study which brain functional domains are more apt to show what changing patterns.

#### 2.4.2. Investigating aging effect on brain functional networks

**Figure 2:**
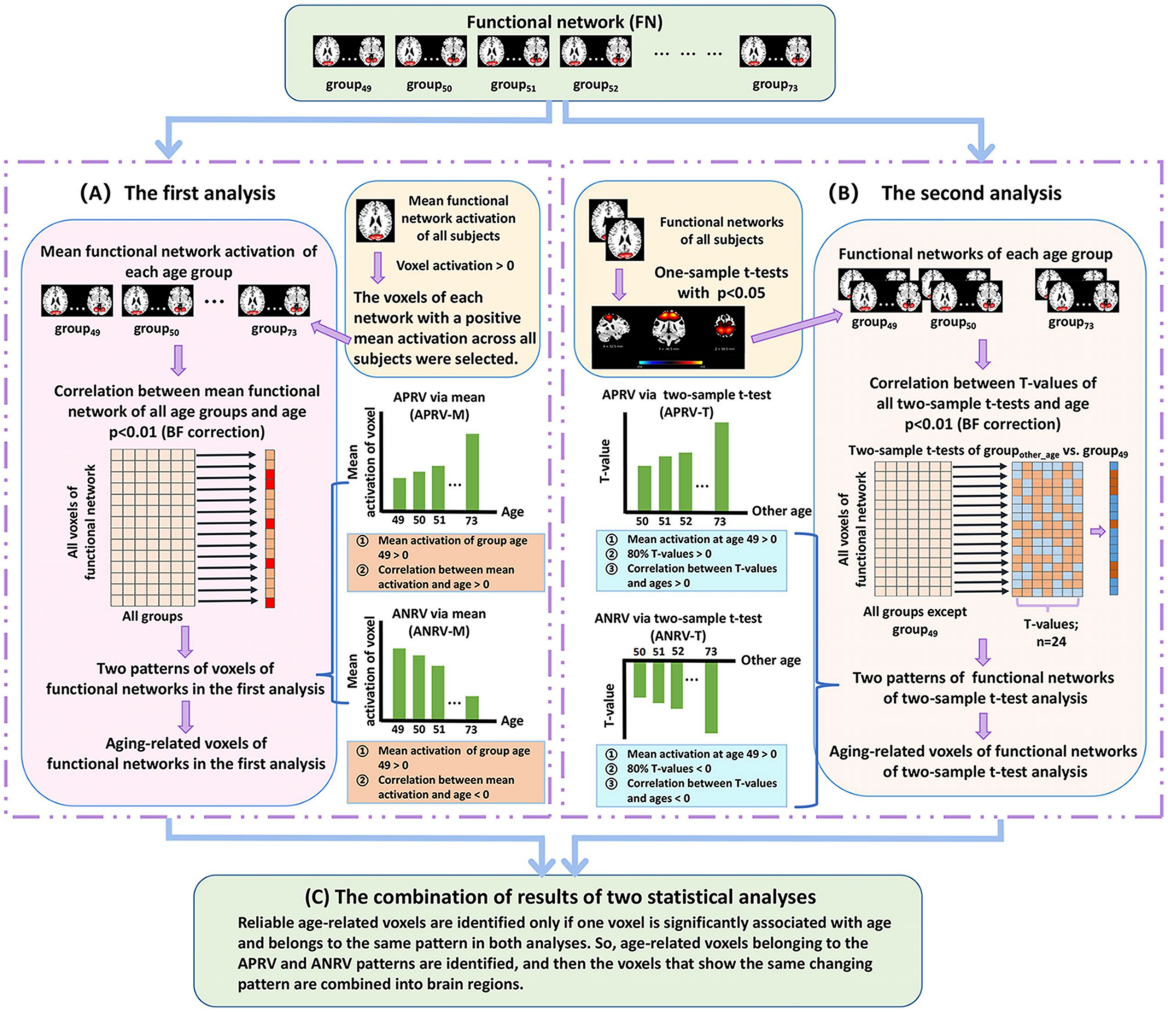
The two-level analysis framework of brain functional network. **(A)** The first analysis. For each voxel of each network that shows positive mean Z-score, we compute voxel’ mean Z-score value in each age group and then compute Pearson correlation between mean values of all groups and ages. After that, each significant age-related voxel passing the correction [*p* < 0.01, Bonferroni (BF) correction] is assessed to judge if it belongs to one of two patterns including the age-positively-related voxel (APRV-M) and the age-negatively-related voxel (ANRV-M). **(B)** The second analysis. For each voxel of each network that significantly shows positive Z-score, we perform two-tailed two-sample *t*-test analysis on its value between each other group and the group of age 49, and then compute Pearson correlation between *T*-values and corresponding ages. After that, each significant age-related voxel passing the correction [*p* < 0.01, Bonferroni (BF) correction] is assessed to judge if it belongs to one of two patterns including the age-positively-related voxel (APRV-T) and the age-negatively-related voxel (ANRV-T). **(C)** The combination of results of two statistical analyses. Reliable age-related voxels are identified only if one voxel is significantly associated with age and belongs to the same pattern in both analyses. So, age-related voxels belonging to the APRV and ANRV patterns are identified, and then the voxels that show the same changing pattern are combined into brain regions.

In a similar manner, we apply a two-level statistical analysis framework (shown in Figure 2) to identify the reliable aging-related brain regions within functional networks. In brief, for each voxel’s Z-score value in each functional network, two types of statistical analysis are performed separately to evaluate its association with the aging and determine its changing pattern, and then the voxel is regarded as being aging-related in case that it shows a significant association with the aging in both analyses, finally its changing pattern is judged accordingly and its property is summarized. After extracting voxels with each changing pattern in each functional network, we further analyze the brain regions each of which includes voxels with the same changing pattern and their related functional domains.

Regarding the first analysis (Figure 2A), since the voxels with positive Z-scores have more important information than that with negative Z-scores in the functional networks obtained from ICA (Du et al., 2015), we only focus on studying the voxels which have positive mean Z-scores across all subjects. For each such voxel in each network, we first average the voxel’s Z-score values in network across the subjects in each age group, and then compute Pearson correlation (denoted by $r_{\mathrm{VoxelMean}\_\mathrm{Age}}$ ) between the mean Z-scores of different age groups (i.e., group_49_ to group_73_) and the ages (i.e., 49–73) to evaluate its relation with aging. For the voxels that are significantly associated with aging, measured by $r_{\mathrm{VoxelMean}\_\mathrm{Age}}$ after the BF correction (*p* < 0.01), we further investigate its aging pattern. Here, each voxel is assessed based on the mean Z-score of group_49_ as well as the correlation between mean Z-score and age (i.e., $r_{\mathrm{VoxelMean}\_\mathrm{Age}}$ ). As such, in the first analysis using the mean Z-score, the defined two changing patterns include the age-positively-related voxel (APRV-M) and the age-negatively-related voxel (ANRV-M). For the APRV-M pattern, the mean Z-score of group_49_ is positive, and the voxel’s mean Z-score across different ages has a positive correlation with age (i.e., $r_{\mathrm{VoxelMean}\_\mathrm{Age}}$ > 0). Regarding the ANRV-M pattern, the mean Z-score of group_49_ is positive, and the voxel’s mean Z-score across different ages has a negative correlation with age (i.e., $r_{\mathrm{VoxelMean}\_\mathrm{Age}}$ < 0).

In the second analysis (Figure 2B), for each functional network, we first perform a right-tailed one-sample *t*-test to select the voxels that show significant positive Z-scores (*p* < 0.05). For each selected voxel, a two-tailed two-sample *t*-test is performed between its Z-score value in group_other-age_ and that in group_49_, and then we utilize 24 *T*-values of all two-sample *t*-tests to calculate its Pearson correlation (denoted by $r_{\mathrm{VoxelT}\_\mathrm{Age}}$ ) with the ages (i.e., from 50 to 73). Next, the voxels, which are significantly aging-related measured by $r_{\mathrm{VoxelT}\_\mathrm{Age}}$ (*p* < 0.01, BF correction), are evaluated using our defined two patterns based on the mean Z-score in group_49_, the *T*-values from the inter-group two-sample *t*-tests, and the correlation $r_{\mathrm{VoxelT}\_\mathrm{Age}}$ . As such, for the second analysis using *T*-values, the two changing patterns are defined as the age-positively-related voxel (APRV-T) and the age-negatively-related voxel (ANRV-T) patterns. In detail, one voxel is regarded as belonging to the APRV-T pattern if its mean Z-score in group_49_ is positive, more than 80% *T*-values are positive, and the *T*-values have a positive correlation with the ages (i.e., $r_{\mathrm{VoxelT}\_\mathrm{Age}}$ > 0). Regarding ANRV-T pattern, the voxel’s mean Z-score of group_49_ is positive, more than 80% *T*-values are negative, and the *T*-values have a negative correlation with the ages (i.e., $r_{\mathrm{VoxelT}\_\mathrm{Age}}$ < 0). Moreover, we calculate the number of voxels that significantly show group difference (*p* < 0.01) in the two-sample *t*-tests between group_other-age_ and group_49_ in each functional network, and then sum up such voxel numbers across all networks to investigate whether the within-network connectivity differences increase along with the increasing age gap.

Based on the results from the above-mentioned two statistical analyses, we identify reliable age-related brain regions in functional networks (Figure 2C). Here, different from the FNC analysis that investigates each FNC, we extract brain regions each of which contains abundant voxels with the same changing pattern for the subsequent study, because obviously single voxel’s connectivity in networks is meaningless. In particular, if one significant age-related voxel belongs to the APRV-M/ANRV-M pattern in the first analysis and the APRV-T/ANRV-T pattern in the second analysis, it is regarded as the APRV/ANRV pattern and its correlation with aging is computed as the mean of $r_{\mathrm{VoxelMean}\_\mathrm{Age}}$ and $r_{\mathrm{VoxelT}\_\mathrm{Age}}$ . In this way, for each functional network, we preserve the voxels that are significantly related to aging after the BF correction in both analyses, and then combine the voxels that show the same changing pattern in both analyses into brain regions. Thus, for each functional network, the brain regions showing the APRV pattern and the brain regions showing the ANRV pattern are extracted, respectively. It is worth pointing out that one network can compose of both brain region in the APRV pattern and brain region in the ANPV pattern, which means that different parts of one network may change in a disparate way. Also, due to different networks could result in brain regions with various sizes, in this study we only maintain brain regions with more than 100 voxels for a further investigation. Finally, within each network, we evaluate each pattern related brain region in terms of its association degree with aging by averaging $r_{\mathrm{VoxelMean}\_\mathrm{Age}}$ and $r_{{\mathrm{VoxelT}}_{\mathrm{Age}}}$ correlations across all included voxels as well as its statistical significance by averaging $p_{\mathrm{VoxelMean}\_\mathrm{Age}}$ and $p_{{\mathrm{VoxelT}}_{\mathrm{Age}}}$ across all included voxels. Furthermore, for each pattern, we sum up the voxel numbers in brain regions that belong to each functional domain.

#### 2.4.3. Investigating joint aging effect on functional network connectivity and functional networks

In addition to investigating the aging effects on the spatial functional networks (i.e., the within-network connectivity) as well as their interactions (i.e., between-network connectivity) separately, we also explore whether the within-network connectivity and the between-network connectivity systematically change during the aging process. Our basic idea is to measure the joint change between each reliable aging-related FNC and the connectivities within the two networks linked by the FNC. For each reliable aging-related FNC, since its changing pattern is already determined, the brain regions that have a consistent changing trend with the FNC in two linked networks are used to evaluate their synergistic aging effect. In our study, three age associations that are from one FNC and two brain regions showing a synergistic change with the FNC in the linked two networks are combined to reflect the synergistic aging degree. Similarly, in order to measure the paradoxical changes between one FNC and its linked two networks, the brain regions that have an opposite changing trend with the FNC in two linked networks are used for computing the mean correlation. As there are in total four changing patterns for one FNC and two changing patterns for one brain region in network, we carefully design the synergistic/paradoxical change measures, formulated in the Equations (1)–(4). Although there are various formulations, the rules are consistent in that if the strengths in both the within-network connectivity and the between-network connectivity consistently increase or decrease along the aging, their changes are considered as synergistic changes, otherwise their changes are taken as paradoxical changes. All these measures are positive values, and the greater synergistic/paradoxical change measure supports the stronger synergistic/paradoxical changes between the FNC and its associated two networks.

In detail, for the APRP FNC and APRV/ANRV regions in functional network (FN), a synergistic/paradoxical change degree is computed as

(1) $$r_{{\mathrm{FNC}}_{\mathrm{APRP}}\;\mathrm{and}\;{\mathrm{FN}}_{\mathrm{APRV}/\mathrm{ANRV}}}=\left(r_{{\mathrm{FNC}}_{\mathrm{APRP}}}+|r_{\mathrm{FN}1_{\mathrm{APRV}/\mathrm{ANRV}}}|+|r_{\mathrm{FN}2_{\mathrm{APRV}/\mathrm{ANRV}}}|\right)/3$$

For the ANRP FNC and ANRV/APRV regions in functional network, a synergistic/paradoxical change degree is computed as

(2) $$\begin{array}{c} r_{{\mathrm{FNC}}_{\mathrm{ANRP}}\;\mathrm{and}\;{\mathrm{FN}}_{\mathrm{ANRV}/\mathrm{ARRV}}}=\left(r_{{\mathrm{FNC}}_{\mathrm{ANRP}}}\_|r_{\mathrm{FN}1_{\mathrm{ANRV}/\mathrm{APRV}}}|\_|r_{\mathrm{FN}2_{\mathrm{ANRV}/\mathrm{APRV}}}|\right)/3 \end{array}$$

For the APRN FNC and ANRV/APRV regions in functional network, a synergistic/paradoxical change degree is computed as

(3) $$\begin{array}{c} r_{{\mathrm{FNC}}_{\mathrm{APRN}}\;\mathrm{and}\;{\mathrm{FN}}_{\mathrm{ANRV}/\mathrm{ARRV}}}=\left(\_r_{{\mathrm{FNC}}_{\mathrm{APRN}}}\_|r_{\mathrm{FN}1_{\mathrm{ANRV}/\mathrm{APRV}}}|\_|r_{\mathrm{FN}2_{\mathrm{ANRV}/\mathrm{APRV}}}|\right)/3 \end{array}$$

For the ANRN FNC and APRV/ANRV regions in functional network, a synergistic/paradoxical change degree is computed as

(4) $$\begin{array}{c} r_{{\mathrm{FNC}}_{\mathrm{ANRN}}\;\mathrm{and}\;{\mathrm{FN}}_{\mathrm{APRV}/\mathrm{ARRV}}}=\left(\_r_{{\mathrm{FNC}}_{\mathrm{ANRN}}}+|r_{\mathrm{FN}1_{\mathrm{APRV}/\mathrm{ANRV}}}|+|r_{\mathrm{FN}2_{\mathrm{APRV}/\mathrm{ANRV}}}|\right)/3 \end{array}$$

Here, the *r* values represent the correlations between FNC (or brain regions in functional network) and age. If no age-related region exists for some specific pattern in the linked functional networks, we set the region’s *r* value to 0 for the calculation. As such, two matrices are obtained to reflect the synergistic and paradoxical changes between reliable aging-related FNCs and brain regions in their linked functional networks, respectively. Since we are mostly interested in what FNCs have strong synergistic or paradoxical changes with networks’ own connectivities, we further summarize the FNCs which linked two networks include brain regions with the similar synergistic/paradoxical change trend. After that, we also investigate the FNCs which linked one of the two networks has the brain region showing the synergistic/paradoxical change with them.

## 3. Results

### 3.1. Aging effect on brain functional network connectivity

**Figure 3:**
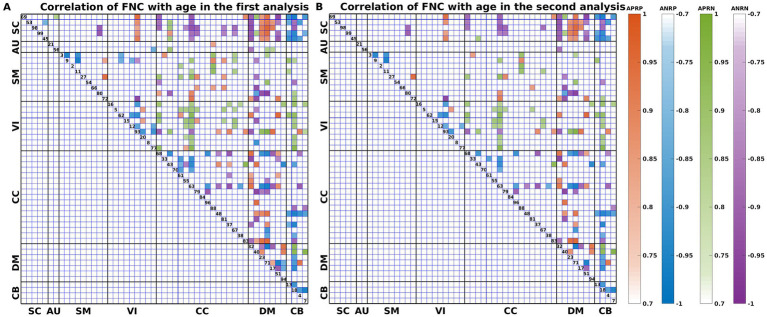
FNC-age correlation of the significant age-related FNCs (*p* < 0.001, BF correction) for **(A)** the first and **(B)** the second analyses. Here, the orange, blue, green, and purple colors represent patterns of APRP-M/T FNC, ANRP-M/T FNC, APRN-M/T FNC, and ANRN-M/T FNC, respectively.

**Figure 4:**
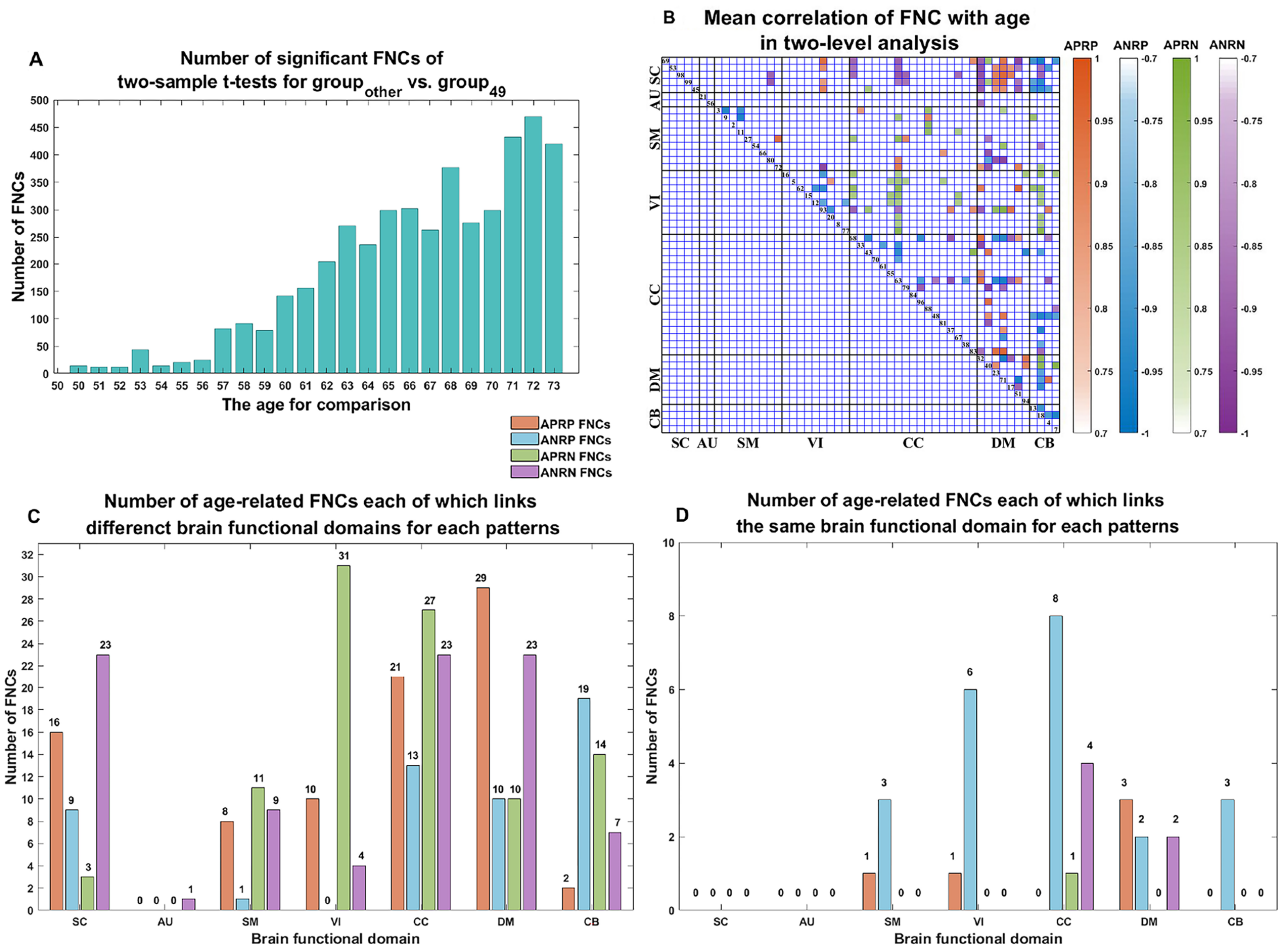
**(A)** Number of significant FNCs of each comparison for the group of other age vs. the group of 49  years old. **(B)** Mean correlation of age-related FNCs for two-level analysis; **(C)** number of age-related FNCs each of which links different functional domains in each of the four changing patterns; **(D)** number of age-related FNCs each of which links the same functional domain in each of four patterns. The orange, blue, green, and purple represent patterns of the age-positively-related positive (APRP) FNC, the age-negatively-related positive (ANRP) FNC, the age-positively-related negative (APRN) FNC, and the age-negatively-related negative (ANRN) FNC, respectively.

As shown in Figure 3, we found that the significant age-related FNCs and their belonged changing patterns were quite consistent between the two types of statistical analysis. Furthermore, Figure 4A shows the number of FNCs that were statistically significant (*p* < 0.01) in comparing each other age group and the group of 49 years using two-sample *t*-tests. It is observed that the inter-group differences increased while the age gap became big. Since the two-level statistical analysis resulted in reliable age-related FNCs for four changing patterns, we show those FNCs of each pattern in Figure 4B. The detailed information of those FNCs can be found in the Supplementary Tables S1–S4. Regarding the APRP FNCs, they were distributed in different brain networks, and included 48 FNCs accounting for 3.96% of all positive FNCs, with a mean FNC-age correlation as 0.89. Regarding the ANRP FNC, they included 48 FNCs accounting for 3.96% of all positive FNCs, with a mean FNC-age correlation as −0.89. Regarding the APRN FNC, they included 49 FNCs accounting for 3.07% of all negative FNCs, with a mean FNC-age correlation as 0.88. Regarding the ANRN FNC, they included 51 FNCs accounting for 3.20% of all negative FNCs, with a mean FNC-age correlation as −0.89.

For each of the four changing patterns, we summarized the age-related FNCs each of which linked different functional domains in Figure 4C, and the age-related FNCs each of which linked the same functional domain in Figure 4D, respectively. Among the 48 APRP FNCs, most of them linked different functional domains. In detail, there were 43 APRP FNCs linking different domains, wherein 29 FNCs of them linked with the DM, and 21 FNCs linked with the CC. For each of the remaining 5 APRP FNCs, it linked the networks within the same domain, wherein 3 FNCs of them linked within the DM. For the ANRP pattern, among 48 FNCs, 26 FNCs linked different domains while the remaining 22 FNCs linked the networks within the same domain. Among the 26 FNCs linking different domains, 19 FNCs of them linked with the CB; among the other 22 FNCs linking the same domain, 8 FNCs linked within the CC and 6 FNCs linked within the VI. For the 49 APRN FNCs, almost all of them (48 FNCs) linked different domains, wherein 31 FNCs of them linked with the VI, and 27 FNCs linked with the CC, while only one 1 FNC linked the networks within the CC domain. For the 51 ANRN FNCs, most of them also mainly involved different domains. In detail, there were 45 FNCs linking different domains, wherein 23 FNCs of them linked with the SC, 23 FNCs of them linked with the CC, and 23 FNCs of them linked with the DM, while other 6 FNCs linked within domains, wherein 4 FNCs of them linked within the CC, and 2 FNCs linked within the DM.

**Figure 5:**
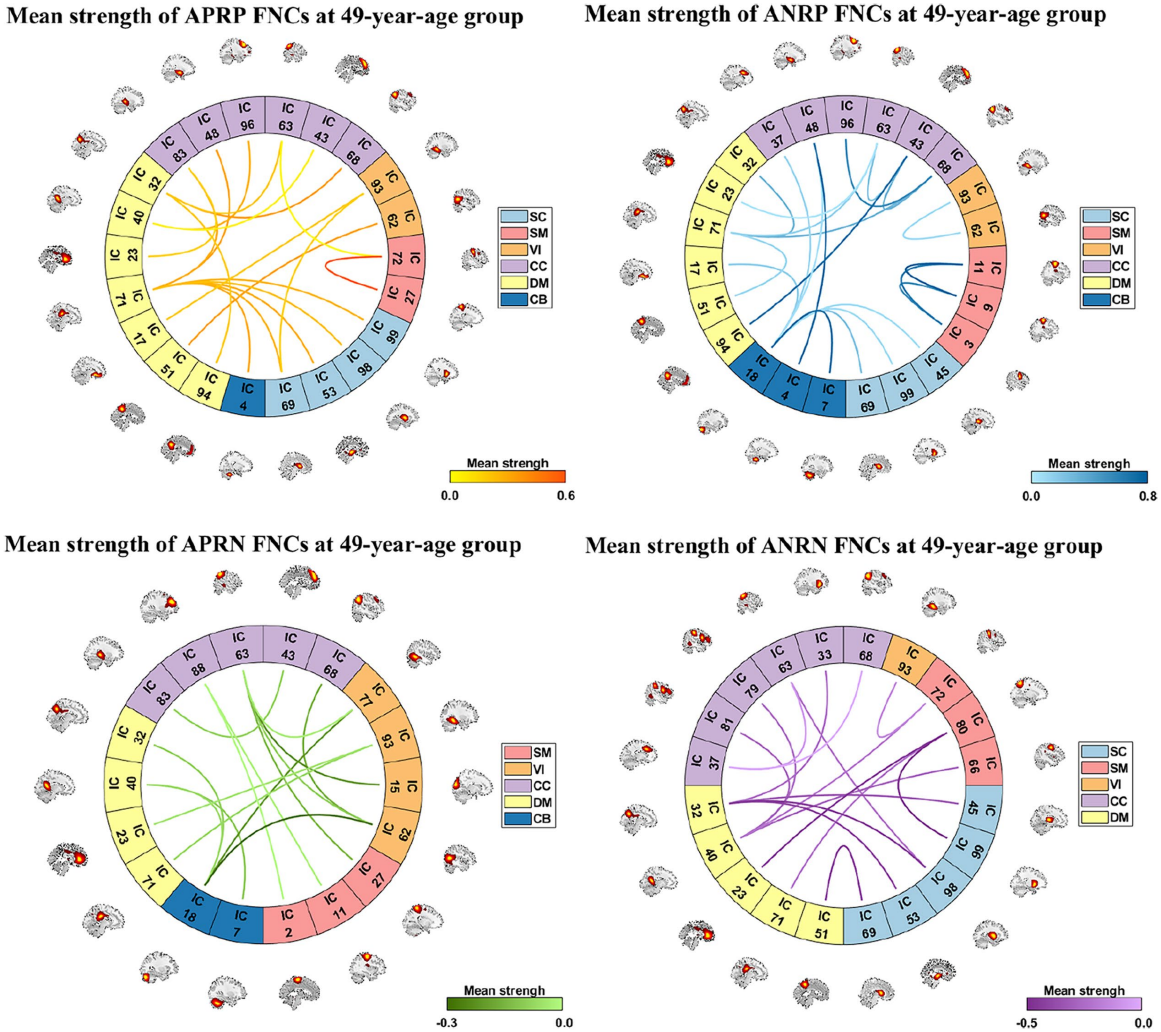
Reliable age-related FNCs each of which has a >0.90 absolute value of the mean correlation (computed by averaging $r_{\mathrm{FNCMean}\_\mathrm{Age}}$ and $r_{\mathrm{FNCT}\_\mathrm{Age}}$ correlations in the two statistical analyses). Here, we separately show these FNCs in four patterns in four subfigures. For each FNC, its mean strength of subjects in the 49-year-age group is shown.

From those reliable age-related FNCs, we further selected the most important FNCs with absolute mean correlation greater than 0.90 for an investigation. Figure 5 displays the mean FNC strength of the 49-year-age group for each of those important FNCs in each pattern. In summary, the APRP pattern comprised 18 FNCs 12 of which linked between the DM and the other domain (CC, VI, or SC); the ANRP pattern comprised 21 FNCs 10 of which linked the same domain (CC, SM, DM, VI, or CB), and 7 of which linked the CB and other domain (SC, CC, or DM); the APRN pattern comprised 15 FNCs, 8 of which linked the VI and the other domain (CC, CB, or DM), and 7 of which linked the CC and the other domain (VI or SM); the ANRN pattern comprised 16 FNCs, 11 of which linked the DM and the other domain (CC, SM, or SC). From the above results, we found that different brain functions could be aging in different changing paths or patterns. Moreover, the aging more affects the FNCs that linked networks belonging to different functional domains compared to the FNCs linking the same domain for each changing pattern, although for the ANRP pattern, many FNCs also came from the same domain.

### 3.2. Aging effect on brain functional network*s*

We observed statistically significant associations between the voxels’ Z-scores in functional networks and the aging in both analyses, and as expected the results were consistent to each other. As mentioned above, the results from two statistical analyses were then combined to identify reliable aging-related brain regions that belong to two changing patterns, respectively, in each functional network. Regarding each of the defined two changing patterns, for each network, we summarized the network’s index, the mean correlation (with age) in the two analyses, the mean value of p in the two analyses, the correlation and value of p in each type of analyses, the voxel number of brain regions in the network, its functional domain, and brain region names according to the automated anatomical labeling (AAL) atlas (Tzourio-mazoyer et al., 2002). Please find the detailed information in Tables 2, 3.

**Table 2:** Information of brain regions in the APRV pattern.

FN ID	Mean corr	Mean *p*	The first analysis		The second analysis		VN	Domain	Region name
			corr	*p*	corr	*p*			
69	0.89	5.52e-08	0.89	2.52e-08	0.89	8.52e-08	163	SC	Caudate_L and Caudate_R
56	0.90	5.58e-08	0.90	2.07e-08	0.90	9.08e-08	333	AU	Temporal_Sup_L and Insula_R
2	0.86	1.87e-07	0.86	8.36e-08	0.85	2.90e-07	146	SM	Precuneus_L and Precuneus_R
11	0.86	1.77e-07	0.87	7.77e-08	0.86	2.76e-07	173	SM	Postcentral_R and SupraMarginal_R
27	0.87	1.74e-07	0.88	1.65e-07	0.87	1.84e-07	341	SM	Parietal_Inf_L and Parietal_Inf_R
15	0.86	2.26e-07	0.86	1.21e-07	0.86	3.32e-07	100	VI	Occipital_Mid_L
79	0.87	1.00e-07	0.88	4.49e-08	0.87	1.56e-07	238	CC	Postcentral_R, Parietal_Inf_R and SupraMarginal_R
81	0.87	1.03e-07	0.87	4.09e-08	0.87	1.65e-07	115	CC	Parietal_Inf_L
23	0.86	1.32e-07	0.87	6.18e-08	0.86	2.03e-07	113	DM	Caudate_L and Caudate_R
71	0.88	7.10e-08	0.89	2.03e-08	0.88	1.22e-07	298	DM	Thalamus_L
7	0.88	8.32e-08	0.88	3.43e-08	0.87	1.32e-07	152	CB	Cerebelum_8_R

FN ID, functional network ID in NeuroMark; corr, correlation value; VN, voxel number.

**Table 3:** Information of brain regions in the ANRV pattern.

**FN ID**	**Mean corr**	**Mean *p***	**The first analysis**		**The second analysis**		**VN**	**Domain**	**Region name**
			**corr**	***p***	**corr**	***p***			
69	−0.88	8.01e-08	−0.88	3.09e-08	−0.87	1.29e-07	149	SC	Putamen_L and Putamen_R
98	−0.89	6.50e-08	−0.90	2.14e-08	−0.88	1.09e-07	151	SC	Putamen_L and Putamen_R
99	−0.88	9.26e-08	−0.88	3.47e-08	−0.87	1.51e-07	186	SC	Pallidum_L and Pallidum_R
21	−0.87	1.65e-07	−0.87	5.88e-08	−0.86	2.72e-07	111	AU	Polandic_Oper_L
56	−0.86	1.64e-07	−0.87	6.70e-08	−0.86	2.62e-07	243	AU	Insula_L and Cingulum_Mid_R
2	−0.87	1.25e-07	−0.88	4.01e-08	−0.87	2.09e-07	185	SM	Supp_Motor_Area_R, Paracentral_Lobule_L and Precentral_R
11	−0.87	1.40e-07	−0.88	5.35e-08	−0.86	2.26e-07	190	SM	Precentral_L
54	−0.88	8.89e-08	−0.88	3.40e-08	−0.87	1.44e-07	104	SM	Frontal_Sup_R and Frontal_Sup_L
20	−0.85	2.35e-07	−0.86	8.89e-08	−0.85	3.80e-07	103	VI	Occipital_Inf_R
33	−0.87	1.27e-07	−0.88	3.79e-08	−0.86	2.15e-07	104	CC	Insula_L and Cingulum_Mid_R
61	−0.86	1.37e-07	−0.87	4.81e-08	−0.85	2.25e-07	138	CC	Temporal_Mid_R
32	−0.90	7.97e-08	−0.91	2.81e-08	−0.89	1.31e-07	155	DM	Precuneus_R
23	−0.88	8.67e-08	−0.89	3.97e-08	−0.88	1.34e-07	163	DM	Cingulum_Ant_L and Cingulum_Mid_L
71	−0.88	8.95e-08	−0.88	3.45e-08	−0.87	1.45e-07	230	DM	Precuneus_R and Cingulum_Post_R
18	−0.88	1.05e-07	−0.89	3.35e-08	−0.87	1.76e-07	170	CB	Celebelum_Crus2_L and Celebelum_Crus2_R

FN ID, functional network ID in NeuroMark; corr, correlation value; VN, voxel number.

**Figure 6:**
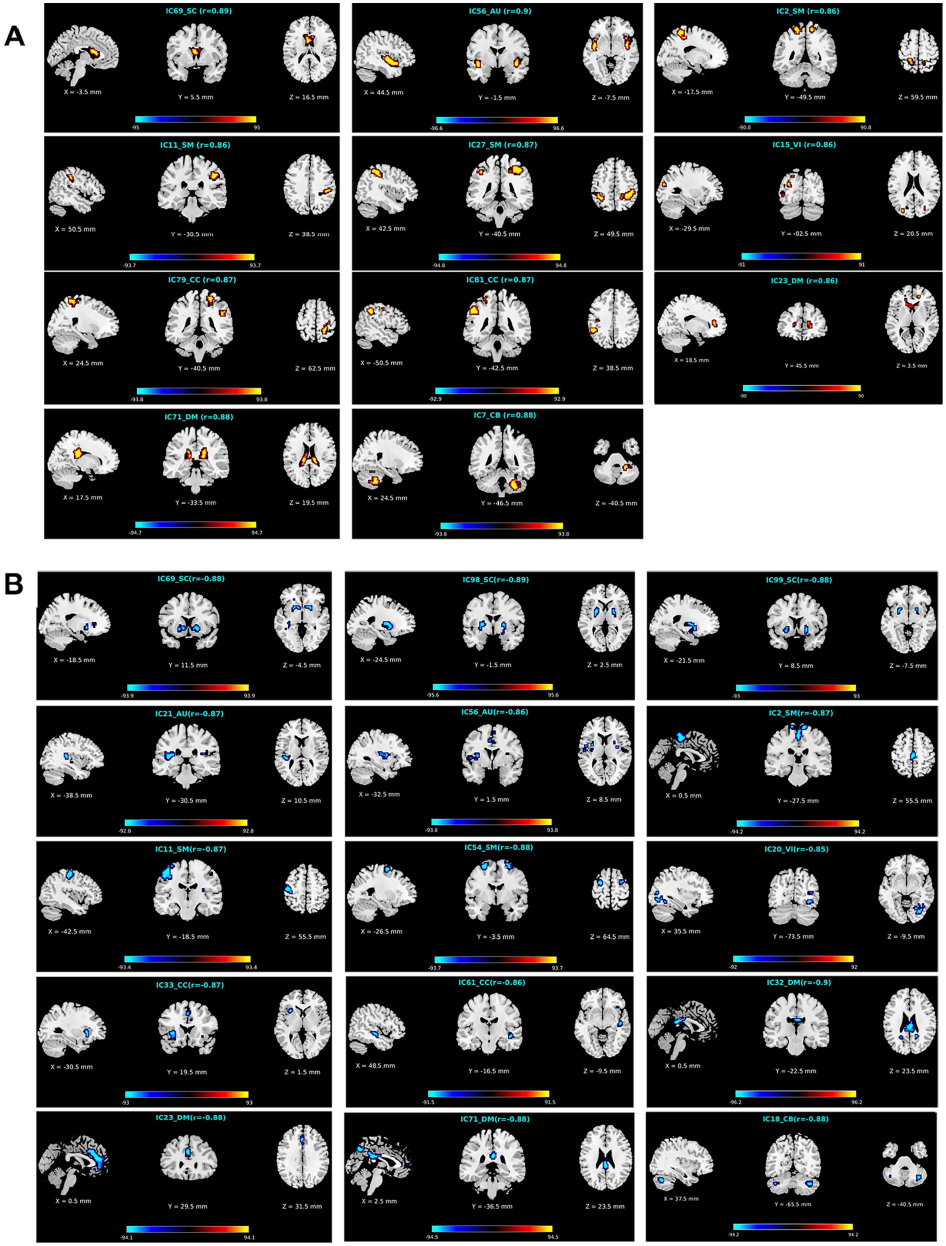
Visualization of functional networks’ regions for **(A)** the APRV pattern and **(B)** the ANRV pattern. In each subfigure, we show the network ID, its functional domain, the mean correlation with age in the brain region, and the correlations with age of all aging-related voxels in the brain region. Note: for a clear display, we multiply the correlation in each voxel by 100.

To further investigate what brain regions in what networks belong to what changing pattern, we demonstrate the identified brain regions for the APRV and ANRV patterns in Figures 6A,B, respectively. For the APRV pattern, 11 functional networks spreading across different domains included brain regions in this pattern, and these regions were all highly correlated with age, with a mean correlation of 0.87. From 15 functional networks, we identified brain regions belonging to the ANRV pattern, with a mean correlation of −0.87. As summarized in the Table 2; Figure 6A, the enhanced within-network connectivity changes primally involved: the caudate nucleus in the SC; superior temporal gyrus and insula in the AU; precuneus, postcentral gyrus, supramarginal gyrus, and inferior parietal region in the SM; middle occipital gyrus in the VI; postcentral gyrus, inferior parietal and supramarginal gyrus in the CC; thalamus, caudate nucleus in the DM; cerebellum inferior in the CB. As displayed in Table 3; Figure 6B, the diminished network within-network connectivity included: the lenticular nucleus putamen and lenticular nucleus pallidum in the SC; rolandic operculum, insula and median cingulate (and paracingulate gyri) in the AU; supplementary motor area, paracentral lobule, precentral gyrus, and superior frontal gyrus dorsolateral in the SM; inferior occipital gyrus in the VI; middle temporal gyrus, insula and median cingulate (and paracingulate gyri) in the CC; precuneus, anterior cingulate (and paracingulate gyri), median cingulate (and paracingulate gyri), precuneus and posterior cingulate gyrus in the DM; cerebellum inferior in the CB. Interestingly, among those functional networks, 6 functional networks from the SC, AU, SM, and DM domains included brain regions in both patterns, and those functional networks involved the caudate (IC69), middle temporal gyrus [MTG] (IC56), paracentral lobule [ParaCL] (IC2), right postcentral gyrus [R PoCG] (IC11), anterior cingulate cortex [ACC] (IC23) and posterior cingulate cortex [PCC] (IC71).

**Figure 7:**
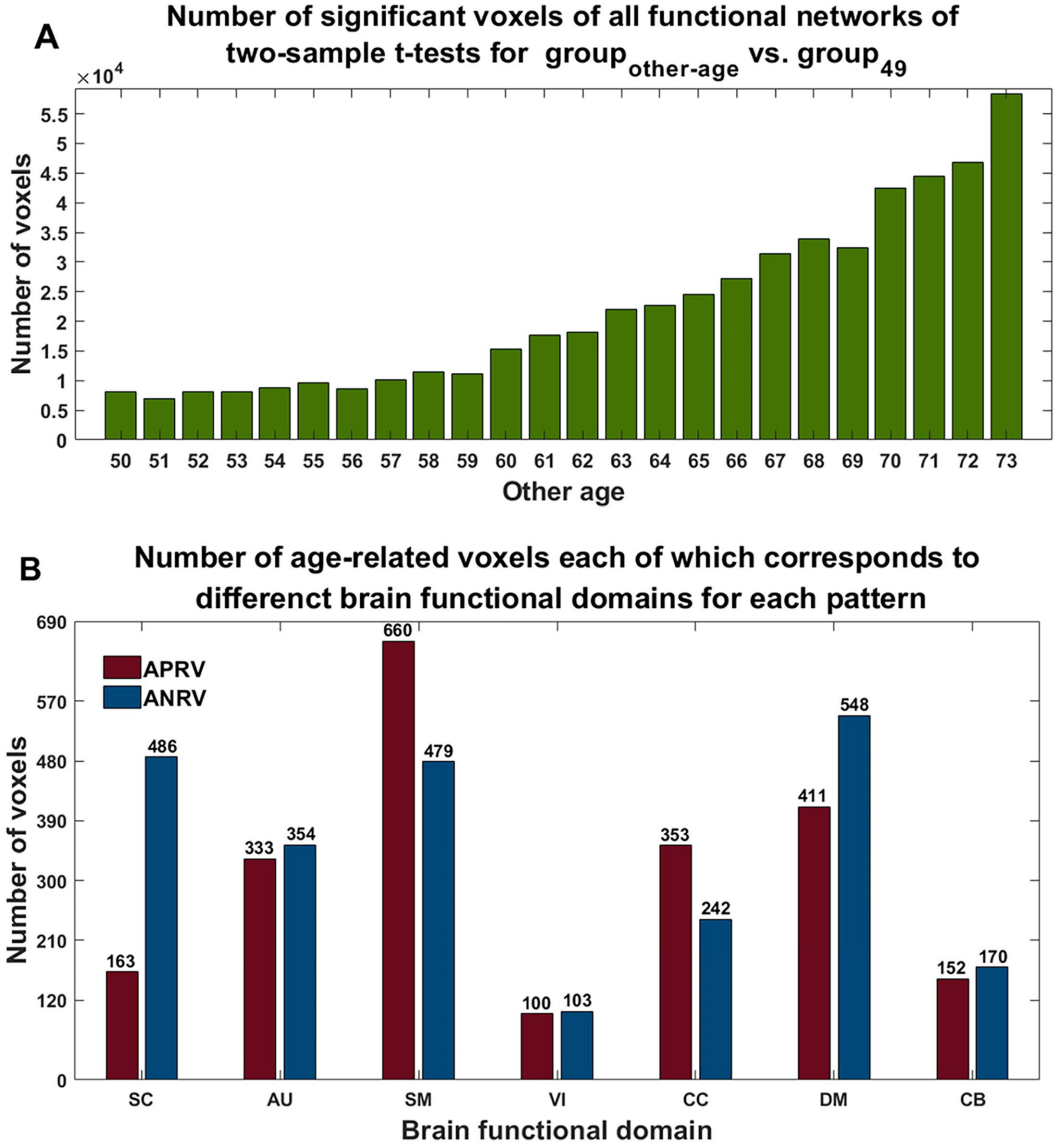
**(A)** Number of significant voxels across all networks in each comparison between the group of other age vs. the group of 49 years old. **(B)** Number of age-related voxels each of which belongs to functional domain in each of two patterns. The red and blue represent the patterns of the age-positively-related positive voxel (APRV) and the age-negatively-related positive voxel (ANRV).

Using two-sample *t*-tests on the voxels’ Z-scores in networks between group_other-age_ and group_49,_ the results (shown in Figure 7A) demonstrate that the number of statistically significant voxels increased greatly while the age gap increased. For each pattern, we calculated age-related voxels in brain regions in functional networks for each functional domain (see Figure 7B). According to the results, the APRV pattern contained a lot of voxels in the SM, DM, and CC networks, with the SM networks showing the most changes in this pattern. The ANRV pattern included many voxels in the DM, SC, and SM networks, while the DM networks showed the most voxels in this pattern. Moreover, although the AU domain only contains two functional networks, both had significant age-related changes.

### 3.3. Joint changes between functional network connectivity and functional networks

**Figure 8:**
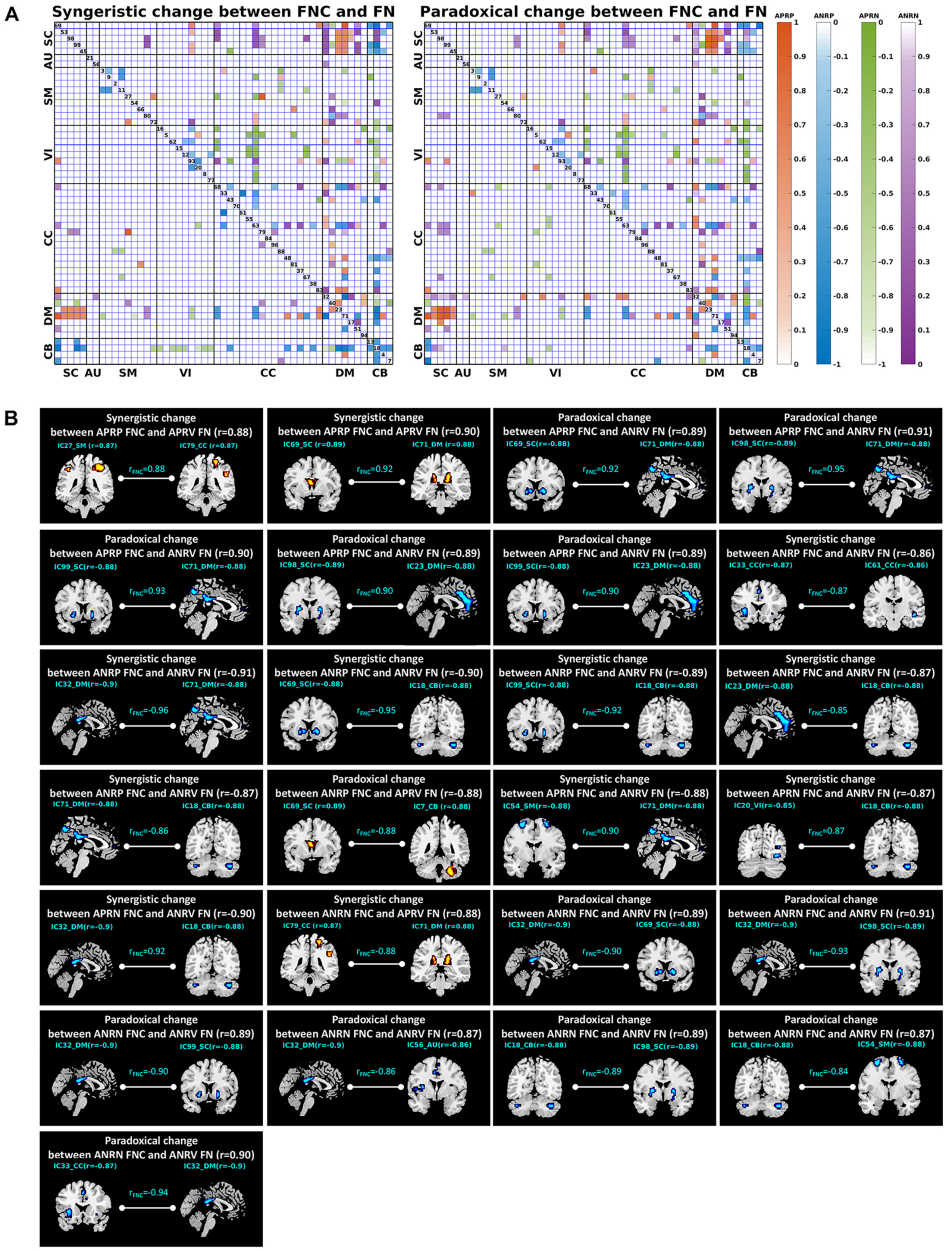
**(A)** Joint change between functional network connectivity (FNC) and functional network (FN). The left and right matrices show the synergistic and paradoxical changes between FNC and FN, respectively. In each matrix, the upper triangular part demonstrates the joint change measures (computed by Equations (1)–(4)) for all reliable age-related FNCs, and the lower triangular part demonstrates the results of the FNCs which had a significant joint change with one or both linked functional networks. The four colors represent the joint change between the FNCs in four patterns and their linked FNs, here the orange, blue, green, and purple represent the FNC patterns of the age-positively-related positive (APRP) FNC, the age-negatively-related positive (ANRP) FNC, the age-positively-related negative (APRN) FNC, and the age-negatively-related negative (ANRN) FNC, respectively. **(B)** Joint changes including the synergistic and paradoxical changes between reliable aging-related FNCs and their linked two functional networks. In each subfigure, we show the FNC-age correlation value, the region-age correlation value in each of the two networks, and the joint change measure computed by Equations (1)–(4).

More importantly, we explored the joint changes including the synergistic and paradoxical changes between reliable aging-related FNCs and their linked functional networks, and show the results in Figure 8; Table 4. For the 48 APRP FNCs, 2 FNCs had a synergistic change with their linked both functional networks, 18 FNCs had a synergistic change with their linked one network, 5 FNCs had a paradoxical change with their linked two networks, and 22 FNCs had a paradoxical change with their linked one network. Regarding the 48 ANRP FNCs, 6 FNCs had a synergistic change with their linked both functional networks, 22 FNCs had a synergistic change with their linked one functional network, 1 FNC had a paradoxical change with their linked both functional networks, and 15 FNCs had a paradoxical change with their linked one functional network. For the 49 APRN FNCs, 3 FNCs had a synergistic change with their linked both functional networks, 17 FNCs had a synergistic change with their linked one functional network, and 18 FNCs had a paradoxical change with their linked one functional network. For the 51 ANRN FNCs, 1 FNC had a synergistic change with its linked both functional networks, 14 FNCs had a synergistic change with their linked one functional network, 7 FNCs had a paradoxical change with their linked both functional networks, and 22 FNCs had a paradoxical change with their linked one functional network. Furthermore, it can be seen from Table 4 that most synergistic changes were present between the reduced FNCs (i.e., the ANRP and APRN patterns showing decreased magnitude in the FNC strengths) and their associated functional networks, and most paradoxical changes were present between the enhanced FNCs (i.e., the APRP and ANRN patterns showing increased magnitude in the FNC strengths) and their associated functional networks.

**Table 4:** Summary of joint changes between functional network connectivity and brain functional network.

Pattern of FNC		APRP FNC (Number: 48)		ANRP FNC (Number: 48)		APRN FNC (Number: 49)		ANRN FNC (Number: 51)	
Pattern of FN		APRV FN (showing a synergistic change with FNC)	ANRV FN (showing a paradoxical change with FNC)	ANRV FN (showing a synergistic change with FNC)	APRV FN (showing a paradoxical change with FNC)	ANRV FN (showing a synergistic change with FNC)	APRV FN (showing a paradoxical change with FNC)	APRV FN (showing a synergistic change with FNC)	ANRV FN (showing a paradoxical change with FNC)
Number of FNCs with both linked networks showing a corresponding change		2	5	6	1	3	0	1	7
Number of FNCs with one linked network showing a corresponding change		18	22	22	15	17	18	14	22
Number of FNCs that link different domains	SC	3	9	5	3	2	1	4	16
	AU	0	0	0	0	0	0	1	1
	SM	2	0	2	2	4	5	1	1
	VI	0	1	1	0	2	5	0	0
	CC	2	1	3	0	0	2	6	1
	DM	15	21	10	9	5	4	3	13
	CB	0	0	13	3	10	1	1	4

FN, functional network; FNC, functional network connectivity.

The upper triangular part of the matrix in Figure 8A demonstrates the joint change measures (computed by Equations (1)–(4)) for all reliable age-related FNCs, and the lower triangular part demonstrates the results of the FNCs which had a significant joint change with one or both linked functional networks. It’s observed that not all reliable age-related FNCs had synergistic or paradoxical changes with their associated functional networks. Furthermore, we summarized the most important joint changes where FNCs had strong synergistic or paradoxical changes with their linked both networks in Figure 8B that includes the FNC-age correlation value, the region-age correlation value in each of the two networks, and the joint change measure for each significant synergistic or paradoxical change. Combined with Table 4, we found that the joint changes manifest in different brain functional domains. For the APRP FNCs, the synergistic changes between them and linked functional networks were mainly related to the DM, and paradoxical changes were significantly associated with the DM and SC. It is seen that the positive FNCs linking the DM and SC showed paradoxical changes with their linked functional networks. While the DM and SC networks’ within-network connectivity showed decreases, their mutual positive connectivity was enhanced along with the aging. Regarding joint changes between the ANRP FNCs and their linked functional networks, synergistic changes were mainly related to the DM and CB, and paradoxical changes mainly involved the DM. In particular, the positive FNCs between the DM and CB that were weakened due to the aging tended to show synergistic changes with their linked functional networks that showed decreased within-network connectivity. Interestingly, from Figure 8B, we also observed that the positive FNCs within both DM and CC showed a synergistic decrease with the DM and CC networks’ within-network connectivity. The synergistic changes of the APRN FNCs and their linked functional networks were mostly related to the CB, while paradoxical changes were scattered across all domains of the brain except the AU. For joint changes of the ANRN FNCs and their linked functional networks, synergistic changes were principally related to the CC, and paradoxical changes are principally related to the DM and SC. Going further to explain the paradoxical changes, the negative FNCs between the DM and SC became stronger (i.e., more negative), while the DM and SC networks’ within-network connectivity were decreased along with the aging. Taken together, the DM networks and the FNCs linking with them were greatly involved into the joint changes. Moreover, the relation between DM and SC networks was heavily affected by the aging process, represented by their enhanced interaction magnitude in both positive and negative connectivity while their own within-network connectivities were decreased.

## 4. Discussion and conclusion

In this paper, we took advantage of a large sample size of fMRI data combined with *a priori*-based ICA method called NeuroMark to explore the significant aging-related changes in both the brain functional networks and their interactions, and more importantly we investigated the joint changes between the within-network connectivity and the between-network connectivity during the aging. Using strict quality control, the subjects in our study had the exactly same sample numbers between genders as well as between different ages, which helped improve the robustness of our findings. In our work, a two-level statistical analysis method was proposed to ensure revealing the reliable aging-related brain changes. In addition, we carefully explored different aging patterns for the within-network and the between-network connectivities, respectively. More importantly, we proposed an effective method to quantify the joint synergistic or paradoxical changes between the within-network and the between-network connectivities. In summary, our work provides new insights into the brain aging of healthy individuals based on a large sample size of data with a strict quality control and advanced analysis methods.

Our study supports that brain aging has a progressive effect on the between-network connectivity (i.e., FNC). By separating the positive and negative connectivity for the analyses, we found that different FNCs present different changing patterns along with the aging, but primary changes involved the DM, CC, VI, and CB domains. Regarding the enhanced connectivities (from the APRP and ANRN patterns), we found that they mainly come from the between-domain and are mainly related to the DM and CC. Specifically, our results support that the positive FNCs which strengths increase (i.e., the APRP pattern) largely involve the DM, and they mainly occur between the DM and other networks such as the CC and SC. On the other hand, for the negative FNCs which strength magnitudes become greater along with aging (i.e., the ANRN pattern), we found that most of them link different domains including the CC, DM, and SC. Although some previous studies have also found enhanced connectivity strengths between the DM and other domains (Betzel et al., 2014; Geerligs et al., 2015; Staffaroni et al., 2018; Zhai and Li, 2019; Zonneveld et al., 2019), our work specifically separates the positive and negative connectivity, thus providing more elaborate information. In addition, numerous studies have shown that as aging, there is an enhancement phenomenon of connectivity among brain networks of different brain domains, and it is mainly embodied in the DM and higher-order brain networks (Jockwitz and Caspers, 2021). As previous studies indicate, the reason why older adults exhibit enhanced functional connectivity of the brain may is a compensatory effect (Cabeza et al., 2002; Grady et al., 2005; Davis et al., 2008; Morcom and Henson, 2018). In addition, we found that weakening of connectivities (from the ANRP and APRN patterns) is primarily associated with the CC, VI, and CB. For the positive FNCs which strengths become weaker along with aging (i.e., the ANRP pattern), they occur not only between different domains, but also within the same domain. Furthermore, the FNCs between the CB and other networks show decreases, while lots of decreased positive FNCs occur within the CC and VI. The findings are supported by some work that also found decreased within-network connectivity in the dorsal attention network and VI (Varangis et al., 2019; Edde et al., 2020; Stumme et al., 2020). For the negative FNCs which strength magnitudes become weaker along with aging (i.e., the APRN pattern), our results suggest that they mainly link the CC and VI with other domains, which is consistent to previous studies (Varangis et al., 2019; Stumme et al., 2020) that found the connections between the VI and SM, CC and other domains are negatively correlated with age. In summary, by analyzing the FNCs, our findings support that the enhancement of FNCs mainly occurs between different domains relating to the DM and CC, while the weakening of FNCs occurs not only between different networks but also within the network, primarily involving the VI, CC and CB. Although some studies have shown that connectivity within the same domain decreases and connectivity between different domains increases (Damoiseaux, 2017), we provide more detailed evidence for aging changes by distinguishing the positive and negative connectivity. Our findings point to a tendency of brain functional networks to become dedifferentiation along with aging, a combined result of increased connectivity between different domains and decreased connectivity within the same domain (Grady et al., 2016), and might also imply that the loss of functional specialization of specific brain networks as the brain ages (Hughes et al., 2020).

In addition to the interaction between networks, we found that many functional networks’ within-network connectivities change gradually with aging. Our results support that the networks with increased within-network connectivity are primarily from the SM and DM, and that with decreased within-network connectivity are mainly in the DM, SC, and SM. We found that the within-network connectivity of the inferior parietal region in the SM and CC gradually increases with aging, which may be supported by previous studies reporting it may affect the ability of the elderly people in implementing sequential behavior (Giller and Beste, 2019). And we also found that the within-network connectivity of caudate in the DM and SC are enhanced with the progressive brain aging, while the damage of the region’s volume is significantly associated with the aging (Verstynen et al., 2012; Bauer et al., 2015) and its function is impaired in common neurodegenerative diseases of older adulthood such as Parkinson’s disease (Pasquini et al., 2019). In our work, the cingulate in the DM, AU, and CC shows an attenuated within-network connectivity, which is relevant to declining cognitive function in the aging population (Pardo et al., 2007). Interestingly, it also often exhibits impairments in receiving and transmitting information in patients with Alzheimer’s disease (Yu et al., 2017). Our study also found a decreased putamen within-network connectivity in the SC, which is consistent with previous work that shows an age association with reduction in putamen volume that is frequently affected by neuropsychiatric and neurodegenerative disorders (Luo et al., 2019). In addition, we found that the frontal gyrus in SM has a decreased within-network connectivity during brain aging, which can be understood as a reason becoming difficult for the attainment of complex behaviors in the elderly (Dippel and Beste, 2015). Besides, we have a unique finding in terms of the within-network connectivity change of insula in aging, while previous studies suggest that self-discrimination in the elderly is linked to insula (Menon and Uddin, 2010; Han et al., 2020). Interestingly, we found that all functional networks in the AU exist significantly aging-related regions, indicating that hearing loss is more common in the elderly (Jayakody et al., 2018; Parthasarathy and Kujawa, 2018; Jafari et al., 2020). Taken together, we found that the aging changes of within-network connectivity are not only related to the DM, SC, and SM, but also involve the AU.

Our study disclosed that there are important joint changes between the within-network connectivity and the between-network connectivity, mostly relating to the DM and SC. Regarding the enhanced positive connectivities (i.e., the APRP FNCs), our research shows that paradoxical changes account for a larger proportion and mainly manifest in the DM and SC, while the synergistic changes are also mainly related to the DM. It is noteworthy that the FNCs linked them gradually increase communication when the within-network connectivity in the DM and SC slowly decreases. Regarding the ANRP FNCs, the difference form enhanced connectivity is that there are more synergistic changes, which are mainly associated with the DM and CB, although the paradoxical changes are likewise mostly associated with the DM. Among the synergistic changes, the connectivity between DM and CB is evident. Their communication is gradually weakened while the within-network connectivity in the DM and CB networks themselves decrease. More interestingly, some FNCs linked within the DM and CC exhibited significant synergistic changes, with the weakened within-network connectivity occurring in both associated functional networks. For the joint changes in the APRN FNCs, there are more synergistic changes, mainly relating to the CB. However, the distribution of the paradoxical change is more various, which almost are present in all brain domains. For the joint changes in the ANRN FNCs, the paradoxical changes show more occupation than the synergistic changes, and they mainly occur in the connectivity with the DM and SC. Interestingly, while the functional networks in the DM and SC showed gradually weakened within-network connectivity during aging, their connectivities with other brain domains become stronger and stronger. In summary, our study clearly highlights that the importance of the DM in brain aging is most evident for joint changes, while the SC is also greatly involved in the joint changes. While some within-network connectivities in the DM are obviously decreased, the connectivity between the DM and other domains are jointly changed but in varied manner. Furthermore, most synergistic changes are present between the FNCs with reduced amplitude and their linked functional networks, and most paradoxical changes are present in the FNCs with enhanced amplitude and their linked functional networks. Our findings may imply that the brain becomes more integrated and functions gradually dedifferentiate in aging, which can further explain the reorganization of the brain with aging (Edde et al., 2020; Jockwitz and Caspers, 2021).

Our study discloses that normal aging even in the absence of neurodegenerative diseases has an important effect on both the whole-brain functional networks (i.e., the within-network connectivity) and the interactions between networks (i.e., the between-network connectivity). More interestingly, the significant joint changes between the within-network connectivity and the between-network connectivity are uniquely found in our work, manifesting the important impairments of default mode networks in the aging process. These findings provide comprehensive evidence for age-related changes in whole-brain. In future, it may be important to identify age-related pathologies in physiological brain aging, in order to establish novel strategies to prevent accelerated pathological brain aging.

## Data availability statement

We used data from the UK Biobank datasets with the agreement of project 34175. The datasets presented in this study can be found in online repositories. The names of the repository/repositories and accession number(s) can be found at: https://www.ukbiobank.ac.uk/.

## Ethics statement

The studies involving human participants were reviewed and approved by Research Tissue Bank (RTB) approval. The patients/participants provided their written informed consents to participate in this study.

## Author contributions

YD applied for the application of the use of the UK Biobank data, designed the whole analysis framework, and proposed the original NeuroMark method. YD and YG performed the analyses and wrote the original draft. All authors contributed to the article and approved the submitted version.

## Funding

This work was supported by National Natural Science Foundation of China (Grant nos. 62076157 and 61703253 to YD), Fund Program for the Scientific Activities of Selected Returned Overseas Professionals in Shanxi Province (to YD), the 1,331 Engineering Project of Shanxi Province of China, the National Institutes of Health (Grant nos. R01MH118695 and R01MH123610 to VC), and the National Science Foundation (Grant no. 2112455 to VC).

## Conflict of interest

The authors declare that the research was conducted in the absence of any commercial or financial relationships that could be construed as a potential conflict of interest.

## Publisher’s note

All claims expressed in this article are solely those of the authors and do not necessarily represent those of their affiliated organizations, or those of the publisher, the editors and the reviewers. Any product that may be evaluated in this article, or claim that may be made by its manufacturer, is not guaranteed or endorsed by the publisher.
