# Supplementary material for: Aging brain shows joint declines in brain within-network connectivity and between-network connectivity: a large-sample study (N > 6,000)
Source: Front Aging Neurosci. 2023 May 18;15:1159054. doi: 10.3389/fnagi.2023.1159054 (PMC10233064; doi:10.3389/fnagi.2023.1159054)
Supplement: Supplementary file 1 [file Data_Sheet_1.docx]

Supplementary Material

Aging Brain Shows Joint Declines in Brain Functional Network Activation and Interaction: A Large-sample Study (N>6,000)

Yuhui Du ^1,2*^, Yating Guo ^1^, Vince D Calhoun ^2^

^1^School of Computer and Information Technology, Shanxi University, Taiyuan, China

^2^ Tri-Institutional Center for Translational Research in Neuroimaging and Data Science (TReNDS), Georgia State University, Georgia Institute of Technology, Emory University, Atlanta, GA, USA

*** Correspondence:**Yuhui Du
[duyuhui@sxu.edu.cn](mailto:duyuhui@sxu.edu.cn)

The file includes Fig. S1-Fig. S3 and Table S1-Table S4.





Fig. S1. The spatial maps of IC1-IC40 in the 100 independent components (ICs) obtained from the GSP dataset.





Fig. S2. The spatial maps of IC41-IC80 in the 100 independent components (ICs) obtained from the GSP dataset.


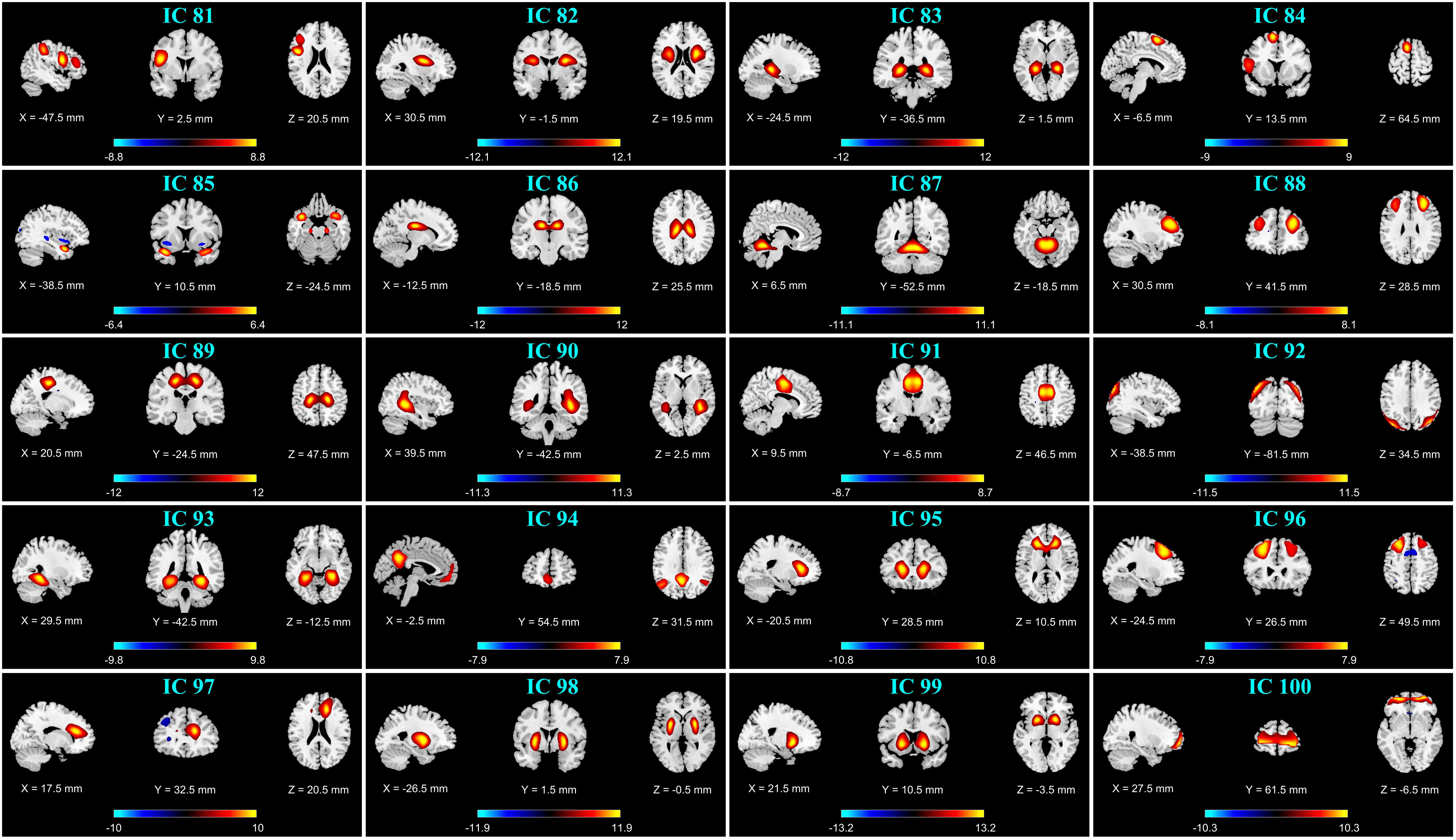


Fig. S3. The spatial maps of IC81-IC100 in the 100 independent components (ICs) obtained from the GSP dataset.

Table.S1. Information of two-level analysis in the APRP pattern.

| **FN**  **ID1** | **FN**  **ID2** | **Mean corr** | **Mean p** | **The first analysis** | | **The second analysis** | | **Mean FNC strength** | **Domain** |
| --- | --- | --- | --- | --- | --- | --- | --- | --- | --- |
|  |  |  |  | **corr** | **p** | **corr** | **p** |  |  |
| 27 | 72 | 0.94 | 7.93e-12 | 0.93 | 7.79e-12 | -0.94 | 8.08e-12 | 0.60 | SM and SM |
| 69 | 93 | 0.94 | 2.18e-11 | 0.94 | 2.56e-12 | -0.93 | 4.11e-11 | 0.25 | SC and VI |
| 53 | 93 | 0.87 | 4.42e-08 | 0.87 | 1.35e-08 | -0.86 | 7.49e-08 | 0.42 | SC and VI |
| 99 | 93 | 0.84 | 2.29e-07 | 0.85 | 5.27e-08 | -0.83 | 4.05e-07 | 0.23 | SC and VI |
| 45 | 93 | 0.90 | 3.49e-09 | 0.90 | 6.80e-10 | -0.89 | 6.31e-09 | 0.30 | SC and VI |
| 5 | 20 | 0.84 | 1.66e-07 | 0.85 | 1.05e-07 | -0.84 | 2.27e-07 | 0.61 | VI and VI |
| 3 | 33 | 0.85 | 1.59e-07 | 0.86 | 3.77e-08 | -0.84 | 2.79e-07 | 0.05 | SM and CC |
| 80 | 63 | 0.88 | 2.06e-08 | 0.89 | 2.88e-09 | -0.87 | 3.84e-08 | 0.21 | SM and CC |
| 72 | 63 | 0.91 | 6.63e-10 | 0.92 | 1.15e-10 | -0.91 | 1.21e-09 | 0.09 | SM and CC |
| 27 | 79 | 0.88 | 1.16e-08 | 0.88 | 7.64e-09 | -0.88 | 1.56e-08 | 0.43 | SM and CC |
| 9 | 88 | 0.89 | 8.99e-09 | 0.89 | 2.30e-09 | -0.88 | 1.57e-08 | 0.08 | SM and CC |
| 66 | 81 | 0.85 | 1.55e-07 | 0.86 | 2.59e-08 | -0.84 | 2.85e-07 | 0.23 | SM and CC |
| 93 | 37 | 0.88 | 1.21e-08 | 0.89 | 3.15e-09 | -0.88 | 2.11e-08 | 0.20 | VI and CC |
| 69 | 83 | 0.87 | 2.38e-08 | 0.88 | 5.33e-09 | -0.87 | 4.22e-08 | 0.72 | SC and CC |
| 93 | 83 | 0.87 | 6.03e-08 | 0.88 | 8.97e-09 | -0.85 | 1.12e-07 | 0.52 | VI and CC |
| 72 | 32 | 0.84 | 2.14e-07 | 0.85 | 1.06e-07 | -0.84 | 3.23e-07 | 0.04 | SM and DM |
| 62 | 32 | 0.90 | 3.17e-09 | 0.91 | 4.90e-10 | -0.89 | 5.84e-09 | 0.21 | VI and DM |
| 68 | 32 | 0.93 | 1.26e-10 | 0.93 | 1.10e-11 | -0.92 | 2.40e-10 | 0.39 | CC and DM |
| 55 | 32 | 0.87 | 2.87e-08 | 0.88 | 5.80e-09 | -0.86 | 5.15e-08 | 0.16 | CC and DM |
| 63 | 32 | 0.92 | 3.11e-10 | 0.93 | 3.30e-11 | -0.91 | 5.89e-10 | 0.25 | CC and DM |
| 43 | 40 | 0.92 | 4.41e-10 | 0.92 | 6.20e-11 | -0.91 | 8.21e-10 | 0.13 | CC and DM |
| 96 | 40 | 0.93 | 5.98e-11 | 0.94 | 3.86e-12 | -0.92 | 1.16e-10 | 0.33 | CC and DM |
| 48 | 40 | 0.86 | 6.17e-08 | 0.86 | 2.44e-08 | -0.86 | 9.90e-08 | 0.19 | CC and DM |
| 53 | 23 | 0.87 | 4.33e-08 | 0.87 | 1.22e-08 | -0.86 | 7.43e-08 | 0.22 | SC and DM |
| 98 | 23 | 0.90 | 2.74e-09 | 0.90 | 6.00e-10 | -0.89 | 4.87e-09 | 0.19 | SC and DM |
| 99 | 23 | 0.90 | 2.15e-09 | 0.90 | 8.03e-10 | -0.90 | 3.50e-09 | 0.31 | SC and DM |
| 45 | 23 | 0.85 | 1.47e-07 | 0.86 | 3.29e-08 | -0.84 | 2.61e-07 | 0.20 | SC and DM |
| 83 | 23 | 0.93 | 2.99e-11 | 0.93 | 1.22e-11 | -0.93 | 4.76e-11 | 0.21 | CC and DM |
| 40 | 23 | 0.87 | 4.04e-08 | 0.87 | 1.47e-08 | -0.86 | 6.62e-08 | 0.34 | DM and DM |
| 69 | 71 | 0.92 | 1.92e-10 | 0.93 | 1.39e-11 | -0.92 | 3.71e-10 | 0.34 | SC and DM |
| 53 | 71 | 0.91 | 5.10e-10 | 0.92 | 1.26e-10 | -0.91 | 8.95e-10 | 0.34 | SC and DM |
| 98 | 71 | 0.95 | 1.07e-12 | 0.95 | 1.49e-13 | -0.95 | 1.99e-12 | 0.31 | SC and DM |
| 99 | 71 | 0.93 | 3.30e-11 | 0.94 | 3.34e-12 | -0.93 | 6.27e-11 | 0.33 | SC and DM |
| 45 | 71 | 0.87 | 5.00e-08 | 0.87 | 1.28e-08 | -0.86 | 8.72e-08 | 0.31 | SC and DM |
| 48 | 71 | 0.92 | 2.08e-10 | 0.93 | 3.70e-11 | -0.92 | 3.78e-10 | 0.39 | CC and DM |
| 37 | 71 | 0.86 | 1.17e-07 | 0.87 | 1.68e-08 | -0.84 | 2.16e-07 | 0.41 | CC and DM |
| 83 | 71 | 0.95 | 8.08e-13 | 0.96 | 1.04e-13 | -0.95 | 1.51e-12 | 0.36 | CC and DM |
| 53 | 17 | 0.85 | 1.65e-07 | 0.85 | 5.94e-08 | -0.84 | 2.70e-07 | 0.53 | SC and DM |
| 98 | 17 | 0.90 | 3.20e-09 | 0.91 | 4.23e-10 | -0.89 | 5.98e-09 | 0.47 | SC and DM |
| 93 | 17 | 0.92 | 1.32e-10 | 0.93 | 1.67e-11 | -0.92 | 2.47e-10 | 0.25 | VI and DM |
| 72 | 51 | 0.90 | 2.80e-09 | 0.90 | 7.32e-10 | -0.89 | 4.87e-09 | 0.15 | SM and DM |
| 62 | 51 | 0.95 | 1.83e-12 | 0.95 | 3.82e-13 | -0.95 | 3.27e-12 | 0.36 | VI and DM |
| 68 | 51 | 0.83 | 3.55e-07 | 0.84 | 1.69e-07 | -0.83 | 5.41e-07 | 0.33 | CC and DM |
| 63 | 51 | 0.87 | 3.21e-08 | 0.87 | 1.16e-08 | -0.86 | 5.25e-08 | 0.23 | CC and DM |
| 32 | 94 | 0.92 | 3.31e-10 | 0.92 | 5.14e-11 | -0.91 | 6.11e-10 | 0.22 | DM and DM |
| 40 | 94 | 0.89 | 7.56e-09 | 0.90 | 9.31e-10 | -0.88 | 1.42e-08 | 0.50 | DM and DM |
| 93 | 4 | 0.89 | 4.95e-09 | 0.90 | 1.07e-09 | -0.89 | 8.84e-09 | 0.47 | VI and CB |

Table.S2. Information of two-level analysis in the ANRP pattern.

| **FN**  **ID1** | **FN**  **ID2** | **Mean corr** | **Mean p** | **The first analysis** | | **The second analysis** | | **Mean FNC strength** | **Domain** |
| --- | --- | --- | --- | --- | --- | --- | --- | --- | --- |
|  |  |  |  | **corr** | **p** | **corr** | **p** |  |  |
| 3 | 9 | -0.94 | 2.30e-11 | -0.94 | 2.15e-12 | 0.93 | 4.38e-11 | 0.46 | SM and SM |
| 3 | 11 | -0.90 | 2.15e-09 | -0.91 | 4.58e-10 | 0.89 | 3.83e-09 | 0.56 | SM and SM |
| 9 | 11 | -0.95 | 1.42e-12 | -0.95 | 4.88e-13 | 0.95 | 2.36e-12 | 0.73 | SM and SM |
| 62 | 12 | -0.88 | 8.46e-09 | -0.89 | 4.25e-09 | 0.88 | 1.27e-08 | 0.35 | VI and VI |
| 16 | 93 | -0.88 | 1.93e-08 | -0.89 | 3.97e-09 | 0.87 | 3.47e-08 | 0.20 | VI and VI |
| 62 | 93 | -0.91 | 2.91e-10 | -0.91 | 2.55e-10 | 0.92 | 3.28e-10 | 0.04 | VI and VI |
| 12 | 93 | -0.84 | 2.45e-07 | -0.85 | 9.48e-08 | 0.83 | 3.95e-07 | 0.39 | VI and VI |
| 93 | 20 | -0.89 | 6.22e-09 | -0.89 | 2.42e-09 | 0.88 | 1.00e-08 | 0.38 | VI and VI |
| 12 | 77 | -0.83 | 4.20e-07 | -0.84 | 1.79e-07 | 0.83 | 6.61e-07 | 0.31 | VI and VI |
| 68 | 43 | -0.90 | 2.73e-09 | -0.91 | 3.20e-10 | 0.89 | 5.15e-09 | 0.25 | CC and CC |
| 33 | 61 | -0.87 | 5.38e-08 | -0.87 | 1.15e-08 | 0.86 | 9.60e-08 | 0.34 | CC and CC |
| 33 | 63 | -0.90 | 3.74e-09 | -0.90 | 6.52e-10 | 0.89 | 6.83e-09 | 0.06 | CC and CC |
| 43 | 63 | -0.92 | 4.01e-10 | -0.92 | 4.17e-11 | 0.91 | 7.59e-10 | 0.12 | CC and CC |
| 70 | 63 | -0.84 | 3.25e-07 | -0.86 | 5.22e-08 | 0.83 | 5.97e-07 | 0.34 | CC and CC |
| 68 | 96 | -0.90 | 1.52e-09 | -0.91 | 4.36e-10 | 0.90 | 2.61e-09 | 0.49 | CC and CC |
| 63 | 96 | -0.87 | 2.99e-08 | -0.88 | 5.13e-09 | 0.86 | 5.47e-08 | 0.38 | CC and CC |
| 63 | 38 | -0.84 | 2.91e-07 | -0.85 | 5.86e-08 | 0.83 | 5.23e-07 | 0.46 | CC and CC |
| 80 | 40 | -0.85 | 1.29e-07 | -0.85 | 7.51e-08 | 0.85 | 1.83e-07 | -0.05 | SM and DM |
| 68 | 23 | -0.89 | 1.03e-08 | -0.90 | 1.26e-09 | 0.88 | 1.94e-08 | 0.16 | CC and DM |
| 63 | 23 | -0.92 | 2.00e-10 | -0.93 | 2.31e-11 | 0.92 | 3.78e-10 | -0.07 | CC and DM |
| 68 | 71 | -0.93 | 9.19e-11 | -0.93 | 1.07e-11 | 0.92 | 1.73e-10 | 0.19 | CC and DM |
| 63 | 71 | -0.95 | 5.65e-13 | -0.96 | 7.26e-14 | 0.95 | 1.06e-12 | 0.01 | CC and DM |
| 67 | 71 | -0.89 | 4.59e-09 | -0.90 | 1.51e-09 | 0.89 | 7.67e-09 | -0.01 | CC and DM |
| 32 | 71 | -0.96 | 2.55e-13 | -0.96 | 4.46e-14 | 0.95 | 4.65e-13 | 0.17 | DM and DM |
| 71 | 51 | -0.95 | 1.13e-12 | -0.95 | 1.25e-13 | 0.95 | 2.13e-12 | -0.01 | DM and DM |
| 43 | 94 | -0.92 | 8.30e-11 | -0.92 | 5.78e-11 | 0.92 | 1.08e-10 | 0.63 | CC and DM |
| 69 | 13 | -0.90 | 4.26e-09 | -0.90 | 5.95e-10 | 0.89 | 7.93e-09 | 0.32 | SC and CB |
| 99 | 13 | -0.85 | 1.46e-07 | -0.86 | 3.97e-08 | 0.84 | 2.53e-07 | 0.27 | SC and CB |
| 45 | 13 | -0.89 | 3.95e-09 | -0.90 | 7.33e-10 | 0.89 | 7.17e-09 | 0.28 | SC and CB |
| 48 | 13 | -0.86 | 6.44e-08 | -0.87 | 1.25e-08 | 0.85 | 1.16e-07 | 0.20 | CC and CB |
| 69 | 18 | -0.95 | 1.02e-12 | -0.96 | 3.97e-14 | 0.95 | 2.00e-12 | 0.09 | SC and CB |
| 99 | 18 | -0.92 | 2.22e-10 | -0.93 | 1.92e-11 | 0.91 | 4.25e-10 | -0.02 | SC and CB |
| 45 | 18 | -0.91 | 3.92e-10 | -0.92 | 8.33e-11 | 0.91 | 7.01e-10 | -0.04 | SC and CB |
| 43 | 18 | -0.86 | 8.22e-08 | -0.86 | 3.52e-08 | 0.85 | 1.29e-07 | 0.44 | CC and CB |
| 48 | 18 | -0.94 | 1.49e-11 | -0.95 | 8.14e-13 | 0.93 | 2.90e-11 | -0.04 | CC and CB |
| 37 | 18 | -0.93 | 4.24e-11 | -0.94 | 2.97e-12 | 0.93 | 8.17e-11 | 0.03 | CC and CB |
| 38 | 18 | -0.83 | 4.72e-07 | -0.83 | 2.78e-07 | 0.83 | 6.65e-07 | 0.37 | CC and CB |
| 23 | 18 | -0.85 | 1.41e-07 | -0.86 | 4.31e-08 | 0.84 | 2.39e-07 | 0.15 | DM and CB |
| 71 | 18 | -0.86 | 7.42e-08 | -0.87 | 1.17e-08 | 0.85 | 1.37e-07 | 0.02 | DM and CB |
| 17 | 18 | -0.91 | 8.43e-10 | -0.92 | 9.83e-11 | 0.90 | 1.59e-09 | 0.06 | DM and CB |
| 13 | 18 | -0.89 | 5.11e-09 | -0.90 | 9.81e-10 | 0.89 | 9.25e-09 | 0.33 | CB and CB |
| 53 | 4 | -0.87 | 1.80e-08 | -0.87 | 1.13e-08 | 0.87 | 2.46e-08 | 0.75 | SC and CB |
| 45 | 4 | -0.85 | 9.35e-08 | -0.86 | 5.01e-08 | 0.85 | 1.37e-07 | 0.63 | SC and CB |
| 48 | 4 | -0.90 | 9.57e-10 | -0.91 | 4.88e-10 | 0.90 | 1.43e-09 | 0.69 | CC and CB |
| 18 | 4 | -0.87 | 4.63e-08 | -0.88 | 6.72e-09 | 0.86 | 8.58e-08 | 0.01 | CB and CB |
| 69 | 7 | -0.88 | 1.01e-08 | -0.89 | 2.24e-09 | 0.88 | 1.79e-08 | 0.32 | SC and CB |
| 48 | 7 | -0.86 | 7.62e-08 | -0.87 | 2.33e-08 | 0.85 | 1.29e-07 | 0.17 | CC and CB |

Table.S3. Information of two-level analysis in the APRN pattern.

| **FN**  **ID1** | **FN**  **ID2** | **Mean corr** | **Mean p** | **The first analysis** | | **The second analysis** | | **Mean FNC strength** | **Domain** |
| --- | --- | --- | --- | --- | --- | --- | --- | --- | --- |
|  |  |  |  | **corr** | **p** | **corr** | **p** |  |  |
| 27 | 68 | 0.87 | 2.67e-08 | 0.88 | 6.70e-09 | -0.87 | 4.67e-08 | -0.24 | SM and CC |
| 16 | 68 | 0.87 | 2.71e-08 | 0.88 | 5.40e-09 | -0.87 | 4.87e-08 | -0.10 | VI and CC |
| 62 | 68 | 0.90 | 1.84e-09 | 0.91 | 2.99e-10 | -0.90 | 3.38e-09 | -0.10 | VI and CC |
| 15 | 68 | 0.85 | 1.87e-07 | 0.87 | 2.38e-08 | -0.84 | 3.51e-07 | -0.17 | VI and CC |
| 45 | 43 | 0.86 | 7.68e-08 | 0.86 | 2.82e-08 | -0.85 | 1.25e-07 | 0.01 | SC and CC |
| 93 | 43 | 0.88 | 1.58e-08 | 0.88 | 4.37e-09 | -0.87 | 2.73e-08 | -0.14 | VI and CC |
| 5 | 55 | 0.84 | 3.24e-07 | 0.85 | 8.29e-08 | -0.83 | 5.65e-07 | -0.01 | VI and CC |
| 15 | 55 | 0.87 | 2.32e-08 | 0.87 | 1.10e-08 | -0.87 | 3.53e-08 | -0.07 | VI and CC |
| 12 | 55 | 0.84 | 2.06e-07 | 0.84 | 1.22e-07 | -0.84 | 2.89e-07 | -0.12 | VI and CC |
| 9 | 63 | 0.86 | 4.56e-08 | 0.87 | 1.67e-08 | -0.86 | 7.45e-08 | -0.11 | SM and CC |
| 27 | 63 | 0.92 | 2.23e-10 | 0.92 | 7.93e-11 | -0.92 | 3.66e-10 | -0.08 | SM and CC |
| 16 | 63 | 0.85 | 2.64e-07 | 0.86 | 3.90e-08 | -0.83 | 4.90e-07 | -0.20 | VI and CC |
| 5 | 63 | 0.87 | 3.20e-08 | 0.88 | 5.55e-09 | -0.86 | 5.84e-08 | -0.11 | VI and CC |
| 62 | 63 | 0.93 | 3.77e-11 | 0.93 | 8.13e-12 | -0.93 | 6.72e-11 | -0.04 | VI and CC |
| 15 | 63 | 0.90 | 2.26e-09 | 0.91 | 3.09e-10 | -0.89 | 4.21e-09 | -0.17 | VI and CC |
| 12 | 63 | 0.85 | 1.50e-07 | 0.87 | 2.10e-08 | -0.84 | 2.78e-07 | -0.27 | VI and CC |
| 20 | 63 | 0.86 | 6.57e-08 | 0.87 | 1.16e-08 | -0.85 | 1.20e-07 | -0.25 | VI and CC |
| 8 | 63 | 0.84 | 2.90e-07 | 0.86 | 4.58e-08 | -0.83 | 5.34e-07 | -0.11 | VI and CC |
| 77 | 63 | 0.93 | 2.65e-11 | 0.94 | 4.44e-12 | -0.93 | 4.86e-11 | 0.02 | VI and CC |
| 5 | 79 | 0.84 | 3.09e-07 | 0.86 | 3.99e-08 | -0.83 | 5.77e-07 | 0.04 | VI and CC |
| 3 | 88 | 0.90 | 2.77e-09 | 0.90 | 8.12e-10 | -0.89 | 4.73e-09 | -0.03 | SM and CC |
| 2 | 88 | 0.92 | 3.28e-10 | 0.92 | 7.77e-11 | -0.91 | 5.79e-10 | 0.05 | SM and CC |
| 11 | 88 | 0.91 | 1.05e-09 | 0.91 | 1.75e-10 | -0.90 | 1.93e-09 | 0.04 | SM and CC |
| 5 | 81 | 0.85 | 2.32e-07 | 0.86 | 3.63e-08 | -0.83 | 4.28e-07 | -0.07 | VI and CC |
| 11 | 67 | 0.85 | 1.25e-07 | 0.85 | 6.80e-08 | -0.85 | 1.83e-07 | -0.16 | SM and CC |
| 15 | 67 | 0.87 | 3.42e-08 | 0.88 | 6.77e-09 | -0.86 | 6.16e-08 | -0.15 | VI and CC |
| 12 | 67 | 0.85 | 2.08e-07 | 0.86 | 5.17e-08 | -0.84 | 3.64e-07 | -0.20 | VI and CC |
| 43 | 83 | 0.94 | 1.77e-11 | 0.94 | 4.04e-12 | -0.93 | 3.14e-11 | -0.04 | CC and CC |
| 69 | 40 | 0.89 | 6.15e-09 | 0.89 | 2.35e-09 | -0.88 | 9.95e-09 | 0.04 | SC and DM |
| 99 | 40 | 0.85 | 1.18e-07 | 0.85 | 6.34e-08 | -0.85 | 1.73e-07 | 0.00 | SC and DM |
| 93 | 23 | 0.91 | 3.85e-10 | 0.92 | 1.06e-10 | -0.91 | 6.64e-10 | 0.01 | VI and DM |
| 3 | 71 | 0.84 | 3.90e-07 | 0.84 | 1.14e-07 | -0.83 | 6.66e-07 | -0.22 | SM and DM |
| 54 | 71 | 0.90 | 3.20e-09 | 0.90 | 5.71e-10 | -0.89 | 5.84e-09 | -0.05 | SM and DM |
| 93 | 71 | 0.96 | 2.10e-13 | 0.96 | 2.42e-14 | -0.96 | 3.96e-13 | 0.04 | VI and DM |
| 16 | 94 | 0.86 | 6.89e-08 | 0.87 | 1.21e-08 | -0.85 | 1.26e-07 | -0.06 | VI and DM |
| 9 | 13 | 0.89 | 7.19e-09 | 0.90 | 1.52e-09 | -0.88 | 1.29e-08 | -0.29 | SM and CB |
| 62 | 13 | 0.89 | 5.17e-09 | 0.90 | 1.04e-09 | -0.89 | 9.30e-09 | -0.23 | VI and CB |
| 72 | 18 | 0.85 | 1.79e-07 | 0.86 | 2.54e-08 | -0.84 | 3.33e-07 | -0.31 | SM and CB |
| 16 | 18 | 0.90 | 2.02e-09 | 0.90 | 6.19e-10 | -0.90 | 3.42e-09 | -0.23 | VI and CB |
| 62 | 18 | 0.91 | 7.64e-10 | 0.92 | 9.54e-11 | -0.90 | 1.43e-09 | -0.22 | VI and CB |
| 15 | 18 | 0.84 | 2.89e-07 | 0.85 | 8.25e-08 | -0.83 | 4.96e-07 | -0.11 | VI and CB |
| 12 | 18 | 0.87 | 3.45e-08 | 0.87 | 1.15e-08 | -0.86 | 5.75e-08 | -0.17 | VI and CB |
| 20 | 18 | 0.87 | 3.06e-08 | 0.87 | 1.09e-08 | -0.86 | 5.03e-08 | -0.20 | VI and CB |
| 8 | 18 | 0.84 | 2.26e-07 | 0.85 | 7.91e-08 | -0.84 | 3.74e-07 | -0.02 | VI and CB |
| 77 | 18 | 0.94 | 9.93e-12 | 0.94 | 2.04e-12 | -0.94 | 1.78e-11 | -0.14 | VI and CB |
| 32 | 18 | 0.92 | 1.67e-10 | 0.93 | 2.14e-11 | -0.92 | 3.12e-10 | -0.02 | DM and CB |
| 40 | 18 | 0.93 | 6.87e-11 | 0.94 | 4.89e-12 | -0.92 | 1.33e-10 | -0.03 | DM and CB |
| 16 | 7 | 0.85 | 1.38e-07 | 0.86 | 3.99e-08 | -0.84 | 2.36e-07 | -0.33 | VI and CB |

Table.S4. Information of two-level analysis in the ANRN pattern.

| **FN**  **ID1** | **FN**  **ID2** | **Mean corr** | **Mean p** | **The first analysis** | | **The second analysis** | | **Mean FNC strength** | **Domain** |
| --- | --- | --- | --- | --- | --- | --- | --- | --- | --- |
|  |  |  |  | **corr** | **p** | **corr** | **p** |  |  |
| 98 | 80 | -0.89 | 7.54e-09 | -0.90 | 1.16e-09 | 0.88 | 1.39e-08 | -0.58 | SC and SM |
| 99 | 80 | -0.90 | 1.58e-09 | -0.91 | 4.05e-10 | 0.90 | 2.75e-09 | -0.56 | SC and SM |
| 72 | 93 | -0.94 | 6.50e-12 | -0.94 | 3.47e-12 | 0.94 | 9.54e-12 | -0.19 | SM and VI |
| 69 | 68 | -0.88 | 1.32e-08 | -0.89 | 1.78e-09 | 0.87 | 2.46e-08 | -0.22 | SC and CC |
| 53 | 68 | -0.86 | 9.87e-08 | -0.87 | 1.99e-08 | 0.85 | 1.78e-07 | -0.28 | SC and CC |
| 98 | 68 | -0.87 | 3.49e-08 | -0.89 | 3.36e-09 | 0.86 | 6.65e-08 | -0.33 | SC and CC |
| 93 | 68 | -0.89 | 8.01e-09 | -0.90 | 9.54e-10 | 0.88 | 1.51e-08 | -0.43 | VI and CC |
| 69 | 63 | -0.90 | 3.01e-09 | -0.91 | 3.12e-10 | 0.89 | 5.70e-09 | -0.32 | SC and CC |
| 53 | 63 | -0.88 | 1.58e-08 | -0.89 | 3.12e-09 | 0.87 | 2.85e-08 | -0.34 | SC and CC |
| 98 | 63 | -0.91 | 5.14e-10 | -0.92 | 5.38e-11 | 0.91 | 9.74e-10 | -0.30 | SC and CC |
| 99 | 63 | -0.85 | 1.71e-07 | -0.86 | 2.55e-08 | 0.84 | 3.17e-07 | -0.26 | SC and CC |
| 93 | 63 | -0.85 | 1.43e-07 | -0.87 | 1.75e-08 | 0.84 | 2.68e-07 | -0.54 | VI and CC |
| 98 | 79 | -0.89 | 5.81e-09 | -0.90 | 1.03e-09 | 0.88 | 1.06e-08 | -0.19 | SC and CC |
| 99 | 79 | -0.86 | 7.92e-08 | -0.87 | 1.27e-08 | 0.85 | 1.46e-07 | -0.28 | SC and CC |
| 79 | 96 | -0.90 | 3.32e-09 | -0.91 | 4.50e-10 | 0.89 | 6.20e-09 | -0.41 | CC and CC |
| 63 | 48 | -0.85 | 1.68e-07 | -0.87 | 2.43e-08 | 0.84 | 3.11e-07 | -0.40 | CC and CC |
| 68 | 37 | -0.91 | 1.06e-09 | -0.91 | 2.64e-10 | 0.90 | 1.86e-09 | -0.08 | CC and CC |
| 63 | 37 | -0.91 | 6.61e-10 | -0.92 | 9.01e-11 | 0.91 | 1.23e-09 | -0.17 | CC and CC |
| 98 | 67 | -0.87 | 1.94e-08 | -0.88 | 9.12e-09 | 0.87 | 2.98e-08 | -0.07 | SC and CC |
| 69 | 32 | -0.90 | 2.49e-09 | -0.90 | 8.98e-10 | 0.89 | 4.08e-09 | -0.50 | SC and DM |
| 53 | 32 | -0.92 | 3.28e-10 | -0.92 | 7.91e-11 | 0.91 | 5.77e-10 | -0.47 | SC and DM |
| 98 | 32 | -0.93 | 1.18e-10 | -0.93 | 1.46e-11 | 0.92 | 2.21e-10 | -0.49 | SC and DM |
| 99 | 32 | -0.90 | 3.62e-09 | -0.91 | 3.16e-10 | 0.89 | 6.92e-09 | -0.43 | SC and DM |
| 45 | 32 | -0.91 | 1.11e-09 | -0.91 | 3.27e-10 | 0.90 | 1.90e-09 | -0.39 | SC and DM |
| 56 | 32 | -0.86 | 6.40e-08 | -0.86 | 3.83e-08 | 0.86 | 8.98e-08 | -0.25 | AU and DM |
| 93 | 32 | -0.90 | 4.17e-09 | -0.91 | 5.08e-10 | 0.89 | 7.83e-09 | -0.28 | VI and DM |
| 33 | 32 | -0.94 | 8.29e-12 | -0.94 | 6.38e-12 | 0.94 | 1.02e-11 | -0.28 | CC and DM |
| 83 | 32 | -0.86 | 9.43e-08 | -0.87 | 1.67e-08 | 0.85 | 1.72e-07 | -0.41 | CC and DM |
| 27 | 40 | -0.85 | 1.70e-07 | -0.86 | 5.05e-08 | 0.84 | 2.90e-07 | -0.23 | SM and DM |
| 66 | 40 | -0.92 | 3.63e-10 | -0.92 | 5.18e-11 | 0.91 | 6.73e-10 | -0.35 | SM and DM |
| 72 | 40 | -0.92 | 2.21e-10 | -0.93 | 3.70e-11 | 0.91 | 4.04e-10 | -0.26 | SM and DM |
| 79 | 40 | -0.95 | 2.11e-12 | -0.95 | 8.35e-13 | 0.95 | 3.39e-12 | -0.33 | CC and DM |
| 81 | 40 | -0.90 | 1.01e-09 | -0.91 | 4.22e-10 | 0.90 | 1.59e-09 | -0.35 | CC and DM |
| 80 | 23 | -0.92 | 3.68e-10 | -0.93 | 3.59e-11 | 0.91 | 6.99e-10 | -0.52 | SM and DM |
| 80 | 71 | -0.95 | 5.80e-13 | -0.95 | 1.34e-13 | 0.95 | 1.03e-12 | -0.35 | SM and DM |
| 79 | 71 | -0.88 | 1.82e-08 | -0.88 | 4.76e-09 | 0.87 | 3.16e-08 | -0.28 | CC and DM |
| 68 | 17 | -0.88 | 1.40e-08 | -0.90 | 1.45e-09 | 0.87 | 2.65e-08 | -0.10 | CC and DM |
| 63 | 17 | -0.89 | 7.42e-09 | -0.90 | 1.10e-09 | 0.88 | 1.37e-08 | -0.34 | CC and DM |
| 32 | 17 | -0.89 | 7.29e-09 | -0.89 | 3.21e-09 | 0.88 | 1.14e-08 | -0.40 | DM and DM |
| 69 | 51 | -0.94 | 4.84e-12 | -0.95 | 6.28e-13 | 0.94 | 9.05e-12 | -0.50 | SC and DM |
| 53 | 51 | -0.84 | 2.21e-07 | -0.85 | 8.21e-08 | 0.84 | 3.60e-07 | -0.47 | SC and DM |
| 99 | 51 | -0.86 | 1.25e-07 | -0.87 | 1.46e-08 | 0.84 | 2.35e-07 | -0.40 | SC and DM |
| 45 | 51 | -0.90 | 2.88e-09 | -0.91 | 4.39e-10 | 0.89 | 5.33e-09 | -0.35 | SC and DM |
| 17 | 51 | -0.89 | 6.53e-09 | -0.90 | 1.04e-09 | 0.88 | 1.20e-08 | -0.39 | DM and DM |
| 53 | 18 | -0.86 | 6.76e-08 | -0.87 | 1.31e-08 | 0.85 | 1.22e-07 | -0.14 | SC and CB |
| 98 | 18 | -0.89 | 9.85e-09 | -0.90 | 1.49e-09 | 0.88 | 1.82e-08 | -0.18 | SC and CB |
| 54 | 18 | -0.84 | 3.19e-07 | -0.86 | 4.76e-08 | 0.83 | 5.90e-07 | -0.37 | SM and CB |
| 83 | 18 | -0.87 | 3.50e-08 | -0.89 | 3.55e-09 | 0.86 | 6.65e-08 | -0.09 | CC and CB |
| 68 | 4 | -0.84 | 3.36e-07 | -0.85 | 5.82e-08 | 0.83 | 6.14e-07 | -0.25 | CC and CB |
| 63 | 4 | -0.89 | 4.73e-09 | -0.90 | 5.95e-10 | 0.89 | 8.86e-09 | -0.37 | CC and CB |
